# Flexural and Fracture Behaviors of Ultra-High-Performance Manufactured Sand Concrete Beams with Steel Fibers and Steel Rebars Based on Acoustic Emission

**DOI:** 10.3390/ma19163531

**Published:** 2026-08-20

**Authors:** Shufu Liu, Yuxing Yang, Peiyan Li, Yue Zhang, Yana Mao, Yubo Jiao

**Affiliations:** 1 Gansu Equity Exchange Group, Lanzhou 730030, China; gsliushufu@sina.com; 2 Key Laboratory of Urban Security and Disaster Engineering of Ministry of Education, State Key Laboratory of Bridge Engineering Safety and Resilience, Beijing University of Technology, Beijing 100124, China; yangyuxing@emails.bjut.edu.cn (Y.Y.); lpyan@emails.bjut.edu.cn (P.L.); 15648109662@163.com (Y.Z.); 3 Gansu Academy of Sciences, Lanzhou 730099, China; maoyana198625@sohu.com

**Keywords:** manufactured sand, ultra-high performance concrete, steel fiber, reinforced beams, flexural performance, acoustic emission, crack classification

## Abstract

**Highlights:**

- Flexural and fracture behaviors of reinforced UHPMC beams are investigated and evaluated.
- Effects of manufactured sand replacement ratios and steel fiber contents are demonstrated.
- AE parameters can accurately identify the cracking of reinforced UHPMC beams during four-point bending tests.
- RA-AF effectively classifies cracks during the damage process of UHPMC beams.

**Abstract:**

The use of manufactured sand (MS) as a substitute for natural sand or quartz sand in the production of ultra-high-performance manufactured sand concrete (UHPMC) represents a critical approach to alleviating the shortage of high-quality aggregates and promoting low-carbon development. However, after steel fibers and steel rebars are introduced into this material system, the synergistic working mechanism and damage evolution characteristics of the resulting ultra-high-performance manufactured sand-reinforced concrete (UHPMRC) beams under flexural loading remain largely unexplored. Acoustic emission (AE) technology, owing to its high sensitivity to the initiation and propagation of microcracks, enables real-time dynamic monitoring of UHPMRC beams throughout the entire process from the elastic stage to fracture failure, thereby providing an effective means to reveal the internal performance degradation law. Accordingly, this study conducted simultaneous AE monitoring on small-scale reinforced beams under four-point bending and investigated the effects of MS replacement ratios (0%, 50%, 100%) and steel fiber contents (1.0%, 1.5%, 2.0%). Results show that UHPMRC beams with 100% MS replacement and 1.5% steel fiber content achieve optimal performance. Compared to 0% MS specimens, those with 100% MS exhibit superior early stiffness, ductility, and flexural capacity due to the combined effects of steel fibers and MS. Beams with 2% steel fiber content experienced fiber clustering, reducing bridging capability and promoting earlier cracking relative to those with 1.5% fibers. AE energy parameters accurately identified cracking and characterized crack propagation in UHPMRC beams. Increasing MS content raised the proportion of shear cracks while reducing tensile cracks. The highest shear signal proportion occurred at 1.0% steel fiber content. These findings provide a valuable reference for the design of sustainable high-performance reinforced-concrete structures using manufactured sand.

## 1. Introduction

Due to its economic, environmental, and logistical advantages, manufactured sand (MS) has been widely employed in various engineering fields such as construction, transportation, and water resources in recent years. MS refers to rock particles smaller than 4.75 mm produced by mechanical crushing and screening after rocks, being sourced from a wide range of raw materials [1,2,3]. With the depletion of natural sand and river sand resources, coupled with increased government regulations on mining activities, the substitution of natural sand by MS has become an inevitable trend in the industry’s development.

Ultra-high-performance concrete (UHPC), a novel cementitious material renowned for its exceptional mechanical properties and superior durability, has garnered considerable attention in the field of civil engineering. It can effectively meet the increasing trends of large-scale and complex civil engineering structures while also fulfilling the societal demands for sustainable development through the advancement of high-performance materials [4,5,6,7]. However, due to high production costs and environmental concerns, UHPC has not been widely adopted in practical construction applications. To achieve economic and environmental sustainability while meeting performance requirements, MS has been explored as a fine aggregate in the preparation of ultra-high-performance manufactured sand concrete (UHPMC), as validated by some researchers [8,9,10,11]. Therefore, advancing the practical application of UHPMC has become a crucial direction.

However, previous studies have predominantly focused on material aspects, such as the influence of MS and stone powder on the mechanical and structural properties of UHPC materials [12,13,14,15]. Donza et al. [16] studied the impact of aggregate morphology on the mechanical properties. The results indicate that the morphology of the aggregate can enhance interlocking effects between particles, thereby improving the overall strength of UHPC, particularly in terms of flexural and tensile strength. Ma et al. [7] validated the feasibility of using stone powder as a partial substitute for cementitious materials. The results demonstrate that the introduction of stone powder contributes to enhancing the rheological properties of UHPC mixtures. Yang et al. [17] investigated the substitution of quartz sand in UHPC with recycled rock powder and found that at an 80% substitution rate, the compressive strength of UHPC surpassed that of conventional UHPC mixes. Moreover, they observed a 42% reduction in unit volume cost. Su et al. [18] investigated the flexural performance of UHPMC beams and found them to exhibit excellent mechanical properties and ductility, and the test value of the beam’s flexural bearing capacity was better when the replacement rate of MS was 50%. Chen et al. [19] found that the replacement ratio of MS had no significant relationship with the tensile strength of UHPMC. Another crucial factor influencing concrete performance is the content of steel fibers. The incorporation of steel fibers can enhance the crack control capability of the concrete matrix and improve its strength [20,21]. Hasgul et al. [22] conducted a study showing that UHPC beams with added steel fibers exhibited an increase in flexural capacity of 23% to 50% compared to fiber-free beams. However, the volume fraction of fibers can significantly influence the performance of concrete. Fiber volume fraction has a notable impact on the workability of concrete. Increasing the volume fraction of fibers can increase the likelihood of heterogeneity and agglomeration in concrete [23,24,25].

Currently, there is a limited amount of literature available regarding the study of rein-forced ultra-high-performance manufactured sand concrete (UHPMRC) beams incorporating MS. Kumar et al. [26] investigated the flexural performance of high-performance reinforced-concrete (HPRC) beams made with sandstone aggregates and silica fume. Their study demonstrated that the mineral fillers in crushed sandstone aggregates and silica fume enhanced the flexural stiffness of HPRC beams, resulting in more uniform stress distribution and increased flexural strength. Liu et al. [27] conducted a feasibility analysis on the incorporation of waste foundry sand in UHPC beams, demonstrating that UHPC beams containing suitable proportions of waste foundry sand achieve satisfactory performance. Overall, current research on UHPMC primarily focuses on material properties, with a lack of experimental studies on structural performance. Material-oriented investigations are insufficient to guide the structural performance of UHPC containing MS, and there remains uncertainty regarding the MS substitution ratio in UHPC [28]. Therefore, further investigation into the structural performance of UHPMRC is imperative for gaining deeper insights.

An increasing number of researchers are employing non-destructive testing techniques to monitor damage in reinforced concrete, enabling comprehensive and multi-angle analyses of failure mechanisms. For instance, acoustic emission (AE) techniques [29,30,31,32,33] are utilized to monitor internal structural damage and surface strain patterns in reinforced concrete beams. Ohtsu et al. [34] employed AE rate monitoring during concrete testing to monitor and characterize internal damage states, establishing correlations between AE activity and microstructural changes within concrete. Jiao et al. [35] employed AE techniques to investigate the reinforcing effects of glass fibers on the damage process of asphalt concrete. They analyzed variations in AE parameters and compared them with strain maps and strain data, thereby cross-validating their findings. Shahzad Ashraf et al. [36] investigated the damage localization and evolution process in polyolefin and steel-fiber-reinforced concrete by AE. AE energy is sensitive to early damage to structures, and different energy levels can be used to distinguish the extent of damage. Cumulative energy changes are used to distinguish the damage process. Correlation analysis is performed on acoustic emission energy and the energy absorption value of the test piece to establish a relationship between the two. Energy combined with the *b*-value and load curve are used to characterize the entire damage process.

Although extensive research has been devoted to the material properties of UHPMC, a systematic understanding of the synergistic working mechanisms and damage evolution characteristics of UHPMRC beams remains conspicuously absent. The morphology and gradation of MS not only govern the matrix density and interfacial transition zone properties but also induce multi-scale coupling effects with steel fiber bridging and rebar bond behavior, which collectively dictate the flexural-shear cracking patterns and stiffness degradation of the beam. From an engineering perspective, the widespread availability and cost-effectiveness of MS must be carefully balanced against construction-related factors, including fiber dispersion uniformity, casting quality, and life-cycle durability, as excessive fiber content may compromise structural reliability through agglomeration. To bridge these gaps, this study aims to: (1) systematically investigate the effects of MS replacement ratios (0%, 50%, and 100%) and steel fiber volume fractions (1.0%, 1.5%, and 2.0%) on the flexural behavior, ductility, toughness, and stiffness degradation of UHPMRC beams via four-point bending tests, thereby identifying the optimal mix design; (2) employ acoustic emission (AE) techniques to capture real-time damage signals throughout the entire process from the elastic stage to final fracture and—in conjunction with energy parameters, *b*-value analysis, and RA-AF classification methods—elucidate the governing mechanisms of MS and steel fibers on crack type evolution and damage modes; and (3) establish intrinsic linkages between MS characteristics and the structural load-bearing response from a material–structure synergy perspective, providing a scientific foundation and design reference for the engineering application of MS in ultra-high performance reinforced concrete structures. Ultimately, this research is expected to offer a technically viable and economically sound pathway for the design of high-performance concrete structures in resource-scarce regions and under low-carbon construction demands.

## 2. Materials and Methods

### 2.1. Specimen Designs

Considering the steel fiber content (1.0%, 1.5%, 2.0%) and MS replacement ratio (0%, 50%, 100%), the shear span to depth ratio of the control specimens was 2.04. Five UHPMRC beams with different parameter combinations were designed and tested in this study. The specimens M100-S1.5, M50-S1.5, and M0-S1.5 were used to evaluate the effect of the MS replacement ratio, whereas M100-S1.0, M100-S1.5, and M100-S2.0 were used to evaluate the effect of steel fiber content. The specimen M100-S1.5 served as the common reference specimen in both comparisons. Table 1 presents the detailed design parameters of the experimental beams.

**Figure 1:**
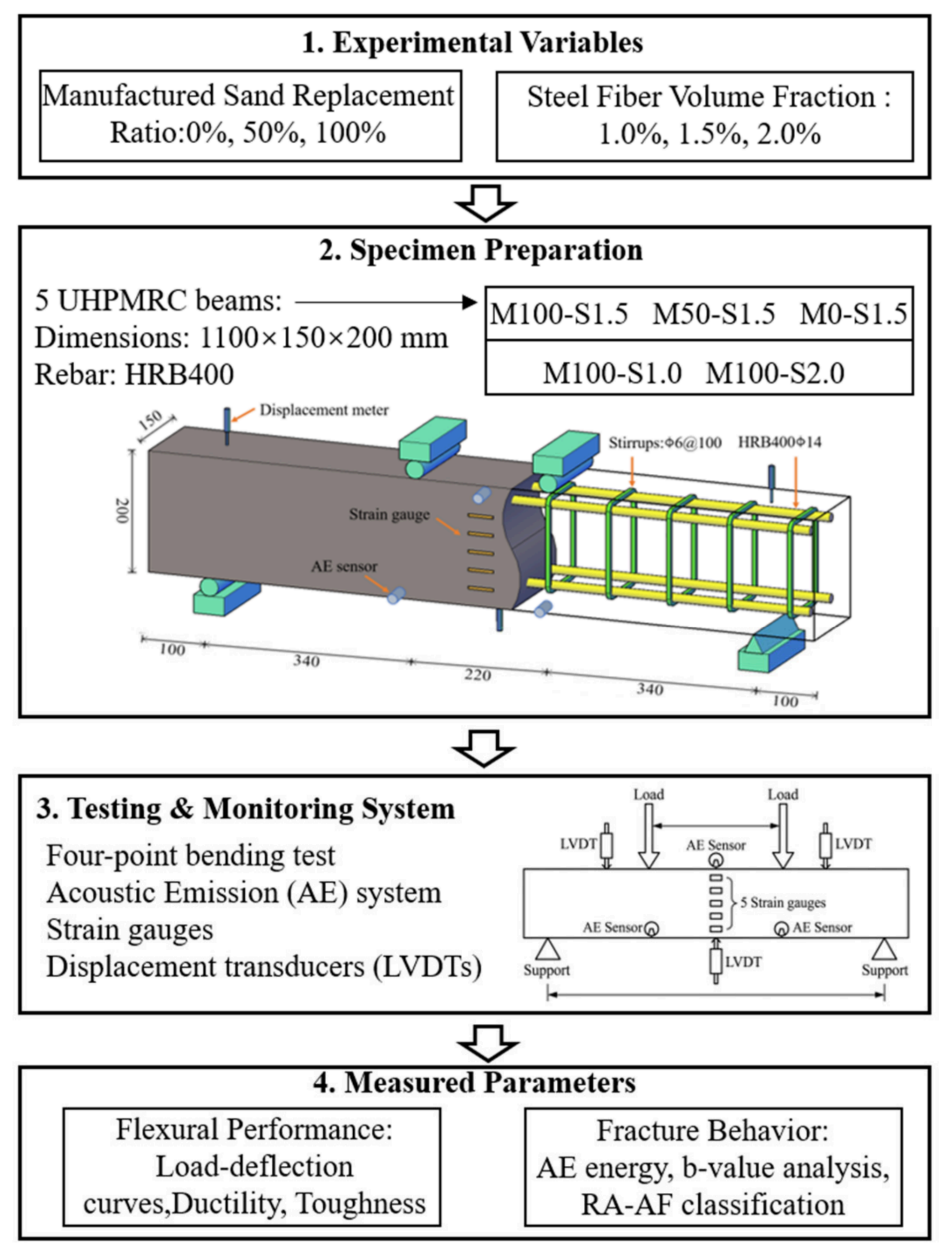
UHPMRC beam test flowchart.

The experimental design dimensions were determined as *l* × *b* × *h* = 1100 × 150 × 200 mm, with each beam containing four HRB400 longitudinal rebars of 14 mm diameter and HRB400 stirrups of 6 mm diameter (Produced by Henan Jiyuan Iron and Steel Group, Jiyuan, China); the thickness of the protective layer was determined as 20 mm. The detailed dimensions of the beam and the test procedure are shown in Figure 1.

### 2.2. Material Properties

#### 2.2.1. Composition of UHPMRC Mixtures

**Figure 2:**
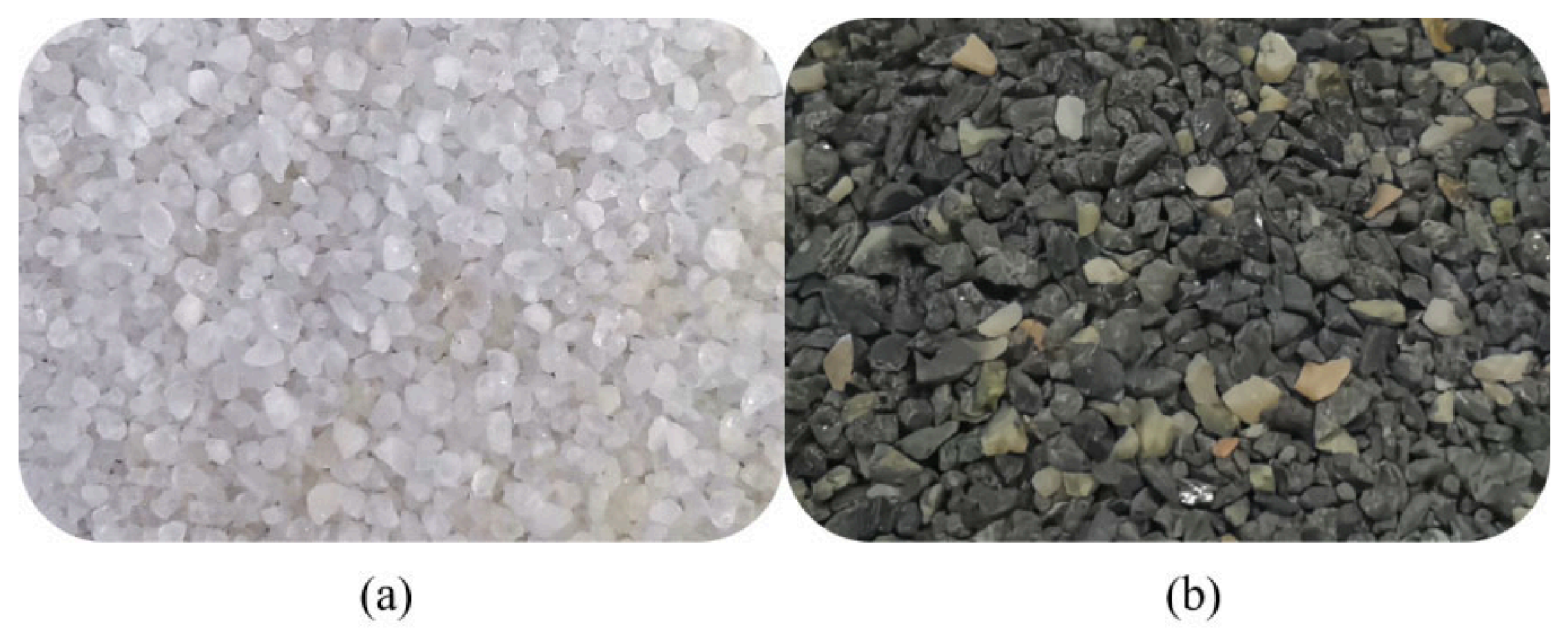
Appearance of (**a**) QS and (**b**) MS.

**Figure 3:**
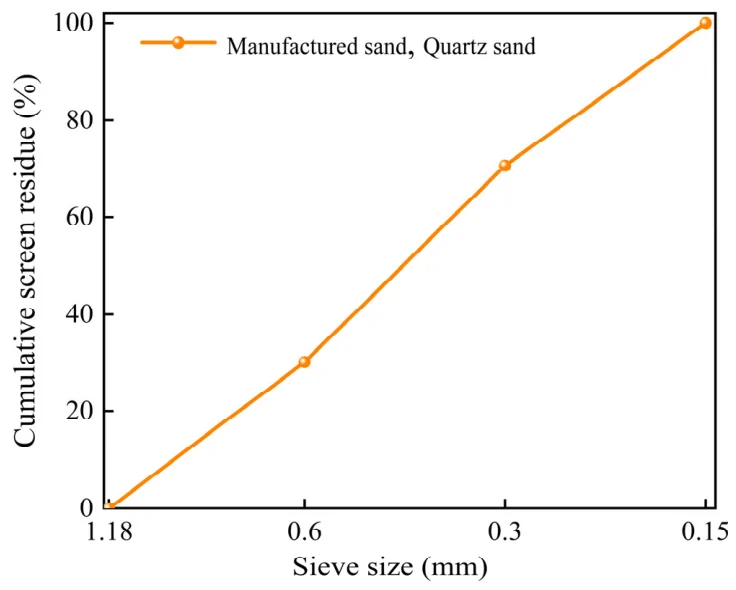
Gradation curves of MS and QS [39].

The UHPMRC mixture used in this experiment primarily consisted of cement, silica fume, quartz powder, quartz sand (QS), MS, steel fibers, and high-performance polycarboxylate superplasticizer. The experiment utilized P.O 52.5 ordinary Portland cement and silica fume (Produced by Shandong Shanshui Cement Group, Weifang, China), which respectively comply with the requirements of “General Portland Cement” (GB 175-2023) [37] and “Silica Fume for Mortar and Concrete” (GB/T 27690-2023) [38]. The experiment employed quartz powder with an average particle size of 10 μm to enhance the density of UHPC. The aggregates consist of QS and MS, as depicted in Figure 2. Through sieve analysis, the particle sizes of MS and QS were controlled within the range of 0.15 mm–1.18 mm, and they exhibited identical particle size distribution curves, as illustrated in Figure 3, consistent with the method used in reference [39]. The experimental fibers used were copper-coated steel fibers with a length of 13 mm, aspect ratio of 70, and tensile strength of 3000 MPa. A superplasticizer based on polycarboxylate ether (Produced by Shanxi Feike Company, Yuncheng, China) with a specific gravity of 1.1 and solid content of 20% (High Performance Water Reducing Admixture, HPWRA) was employed to enhance the flowability of the mixtures. Table 2 presents the mixture proportions for five UHPMRC beams, all having a water-to-binder ratio of 0.16.

#### 2.2.2. Material Property Test

During the fabrication of the test beams, three standard cubic specimens measuring 100 × 100 × 100 mm were prepared for each condition. According to the “Standard Test Methods for Mechanical Properties of Ordinary Concrete” (GB/T50081-2019) [40], compressive strength tests were conducted, and detailed data are presented in Table 3.

Three specimens of each type of reinforcing steel, each measuring 500 mm in length, were reserved for the test beams. Tensile tests on the reinforcing steel were conducted according to “Metallic materials—Tensile testing—Part 1: Method of test at room temperature” (GB/T 228.1-2021) [41], measuring the yield and ultimate tensile strengths of longitudinal and stirrup reinforcement used. Detailed data are provided in Table 4.

### 2.3. Specimen Preparation

**Figure 4:**
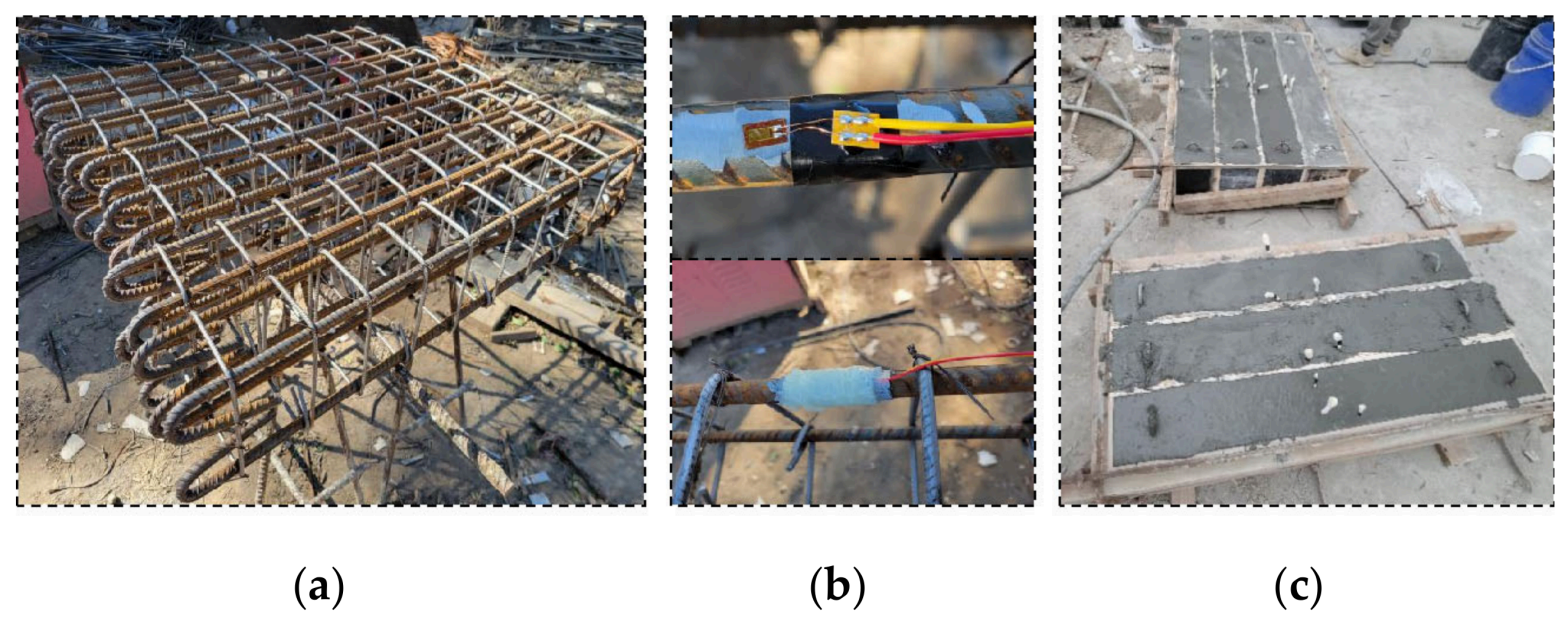
Diagram of the pouring process of the test beam: (**a**) Binding steel cage; (**b**) Pasting strain slice; (**c**) UHPMRC pouring.

The fabrication of test beams involved assembling the steel reinforcement cage, attaching strain gauges to the reinforcement, constructing the formwork, casting the UHPMRC beams, demolding, and curing. During the installation of strain gauges, BX120-30AA strain gauges were positioned on the longitudinal reinforcement at the midspan. Strain gauges were tested for compliance using a digital universal meter, and epoxy resin was applied to waterproof and protect the strain gauges against corrosion. The completed reinforcement cage after binding and the arrangement of strain gauges on the reinforcement are shown in Figure 4a,b. During casting, steel fibers were slowly added to prevent clumping and ensure uniform distribution throughout the mixture. After completion of casting, the test beams are shown in Figure 4c. After allowing it to stand at room temperature for one day until the initial setting of UHPMRC, the formwork was removed, and the specimen was transferred to a standard curing chamber at 95% relative humidity and 20 °C for 28 days.

### 2.4. Test Setup and Instrumentation

To measure the stress variation in the reinforcement during loading, strain gauges were adhered to the bottom longitudinal reinforcement at midspan. Additionally, to investigate the strain distribution along the cross-sectional height of the concrete beam, five strain gauges (BX120-50AA) were evenly spaced at 30 mm intervals along the midspan location on the beam’s side surface. At the midspan and above each support point of the test beam, a displacement transducer was positioned to measure deflection, calculated by subtracting support displacements from midspan displacement. Additionally, the front and back faces of the test beam were uniformly coated with white latex paint. Grid lines spaced 100 mm apart were drawn to facilitate crack observation and highlight crack patterns.

**Figure 5:**
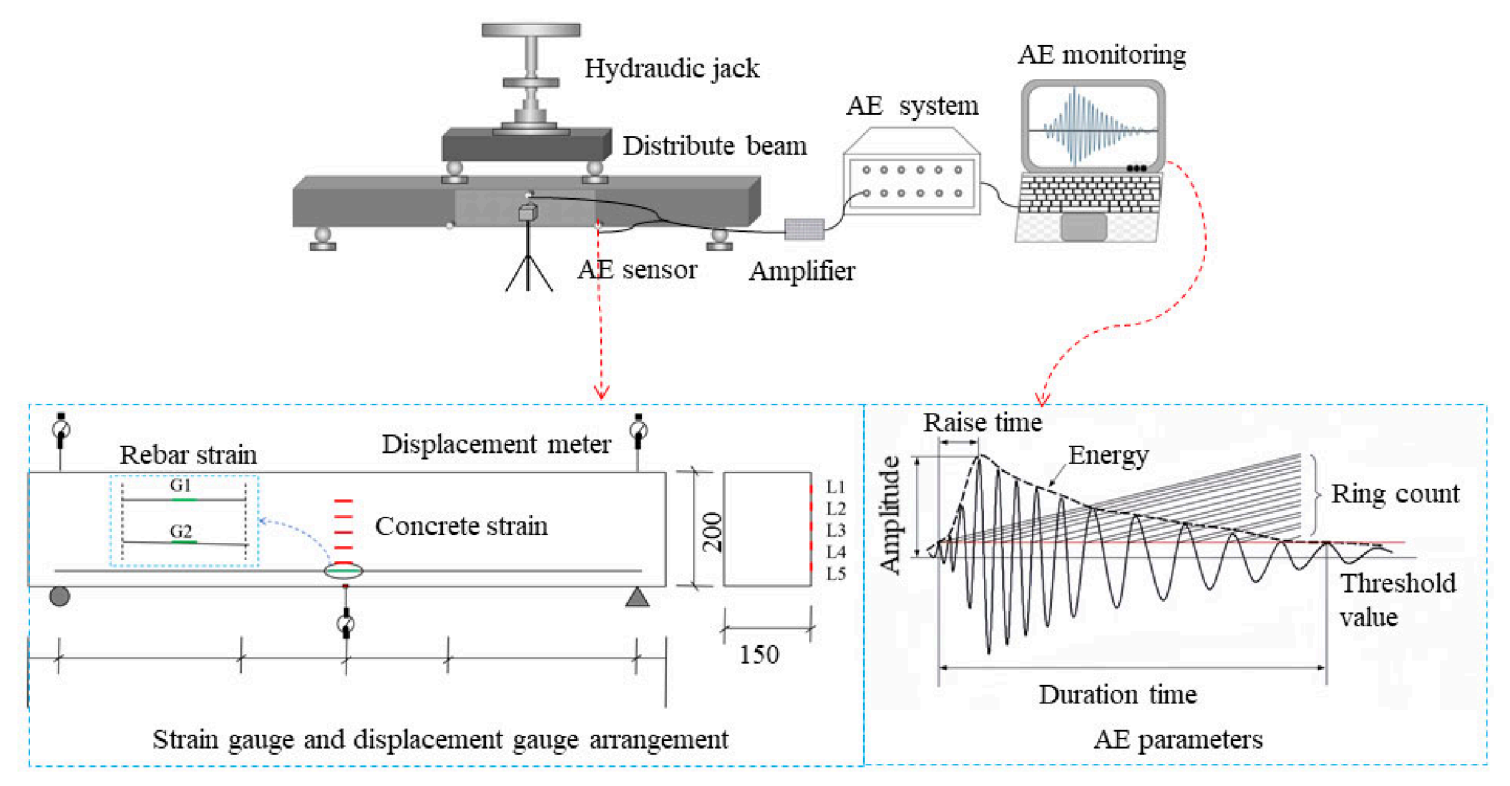
Diagram of the test setup system.

Figure 5 illustrates the experimental setup for testing the entire system. Hydraulic jacks were employed in this experiment to conduct bending tests on all beams. A two-point symmetric loading system was employed under load control conditions. Initially, the loading followed a load-control mode with increments set at 5 kN per stage. After the specimen cracked, the loading increments were adjusted to 15 kN per stage and held for 2 min. Upon reaching 80% of the estimated load capacity, the loading increments were reverted to 5 kN per stage until failure of the specimen. Additionally, an AE device was utilized to monitor crack propagation, ensuring synchronized initiation and termination of all test systems.

### 2.5. Flexural and Fracture Parameters

#### 2.5.1. Ductility, Flexural Toughness and Energy Absorption Capacity

The ductility of UHPMRC beams refers to their ability to resist deformation beyond the elastic stage, which can be quantitatively assessed using a ductility index. This study employs deflection ductility to characterize the ductility of UHPMRC beam structures [42], as shown in Equation (1).

(1) $$\mu =\frac{\Delta_{p}}{\Delta_{y}}$$

where Δ*_p_* represents the mid-span deflection at peak load; Δ*_y_* represents the mid-span deflection at yield load.

Energy absorption is a critical characteristic for assessing the fracture of entire structural components. The ability to absorb energy can be estimated by calculating the area under the load-displacement curve [43].

Ductility is also related to energy absorption. This study evaluates the flexural ductility of UHPC beams using the equivalent initial flexural strength *f_e,m_* and initial energy factor *E_m_*, as shown in Equations (2) and (3) [44].

(2) $$f_{e,m}=\frac{\Omega_{m}L}{\delta_{m}bh^{2}}$$

(3) $$E_{m}=f_{e,m}\times \frac{\delta_{m}}{L}=\frac{\Omega_{m}}{bh^{2}}$$

where *f_e,m_* is designed to adjust for the enhanced bending strength contributed by fiber reinforcement in the material. *E_m_* denotes the material’s energy dissipation capacity per unit volume, which directly reflects the toughness of UHPC. *δ_m_* represents the mid-span deflection corresponding to the peak load. Due to the absence of data collection during the load descent phase, residual toughness analysis was not conducted.

#### 2.5.2. Stiffness Degradation

**Figure 6:**
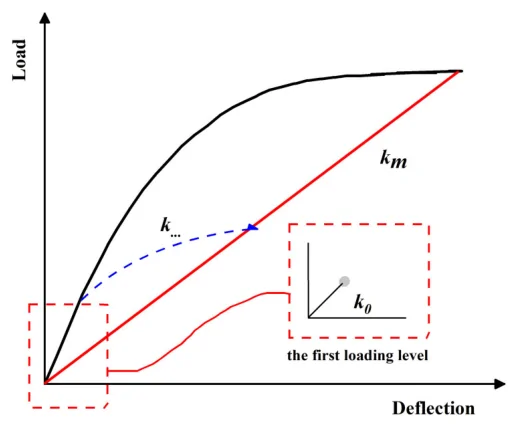
Schematic diagram of secant stiffness.

The experimental analysis of beam stiffness degradation processes was conducted based on a load–deflection curve secant methods [45], with a schematic representation shown in Figure 6. The parameter k represents the stiffness of specimens under different conditions. The initial stiffness of the test beams was taken as the stiffness during the first loading stage.

### 2.6. Acoustic Emission

The AE test aimed to capture stress waves released from internal microcracks in the beam during mechanical testing. A twelve-channel data acquisition system was utilized, employing Vaseline as a coupling agent to maintain proper contact between sensors and specimens. A threshold of 45 dB was applied to mitigate background and mechanical noise. In this experiment, three AE sensors were mounted on the side surface of each beam (*l* × *h* = 1100 × 200 mm). Sensor 1 was placed at the midspan of the beam, 150 mm above the bottom surface. Sensor 2 was positioned 150 mm to the left of the midspan and 50 mm above the bottom surface. Sensor 3 was arranged symmetrically with respect to Sensor 2, 150 mm to the right of the midspan and also 50 mm above the bottom surface. This arrangement was adopted to monitor AE activity in the flexural region and the adjacent shear spans, while maintaining a symmetric sensor layout for the tested beams. The acoustic emission system and its fundamental parameters are illustrated in Figure 5. AE characteristic parameters are closely associated with internal damage in concrete beams, serving as indicators of concrete damage, with energy and cumulative energy being particularly valuable metrics.

The rise angle (RA) is defined as the ratio of rise time to amplitude, while the average frequency (AF) is defined as the ratio of count to duration. RA-AF can be used to distinguish crack types. Typically, high RA and low AF are considered shear cracks, while low RA and high AF are considered tensile cracks.

## 3. Flexural Performance of Reinforced UHPMRC

### 3.1. Failure Mode and Crack Pattern

**Figure 7:**
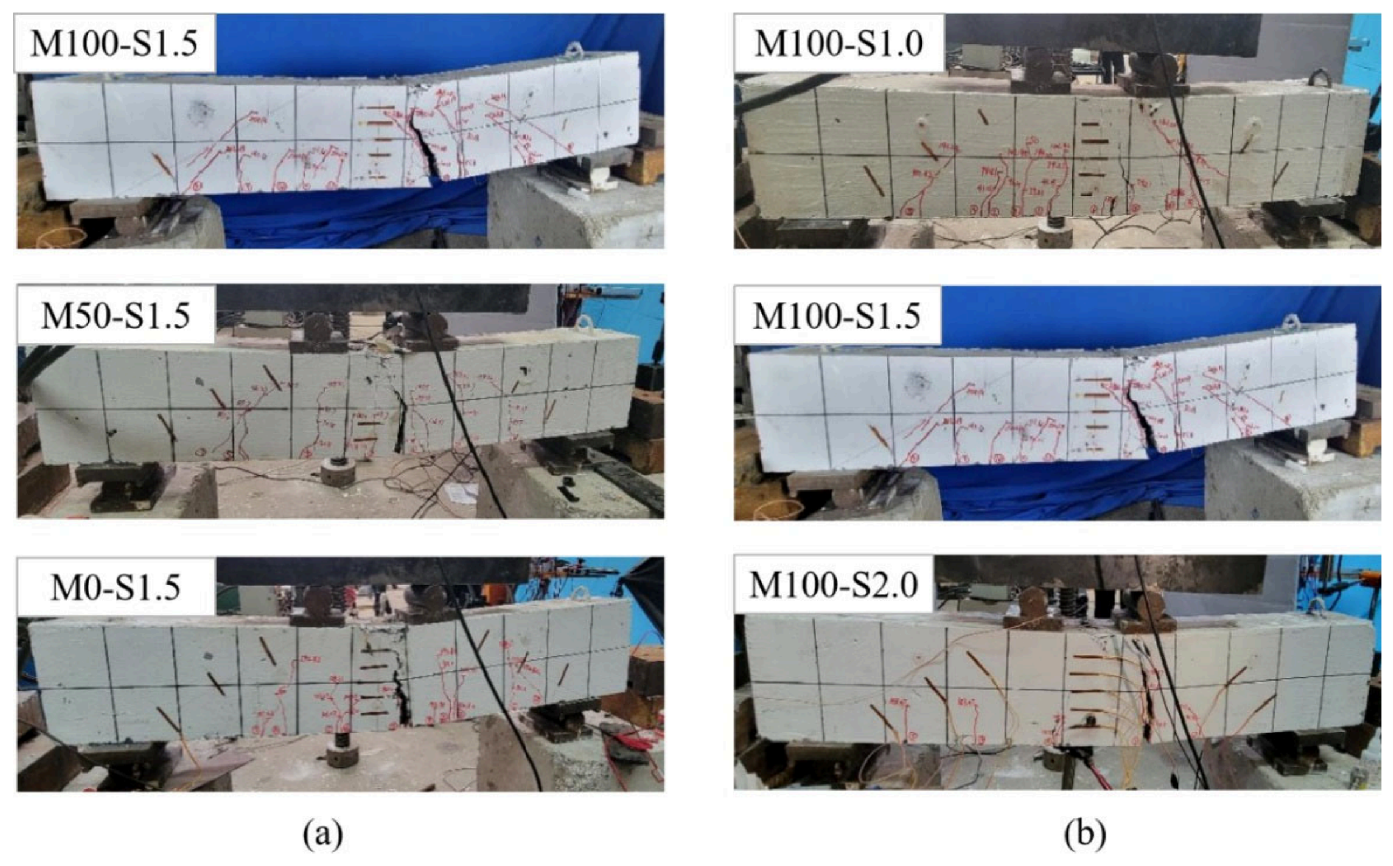
UHPMRC beam failure modes: (**a**) replacement ratios of different MS (**b**) different steel fiber contents.

Figure 7 illustrates the failure modes and crack distribution of the specimens. All test specimens exhibited flexural failure. Tensile failure was observed in the steel-reinforced UHPMRC beam M100-S1.5, M100-S1.0, M100-S2.0, characterized by the fracture of the bottom HRB400 longitudinal reinforcement. However, the compressive zone of the UHPMRC at the top did not reach its ultimate compressive strain and remained undamaged. Compression failure was observed in test beam M0-S1.5 and M50-S1.5, showing that the tensile longitudinal bars were not broken and the concrete in the top compression zone was peeled off. The failure modes of UHPMRC beams varied slightly with different MS replacement ratios.

**Figure 8:**
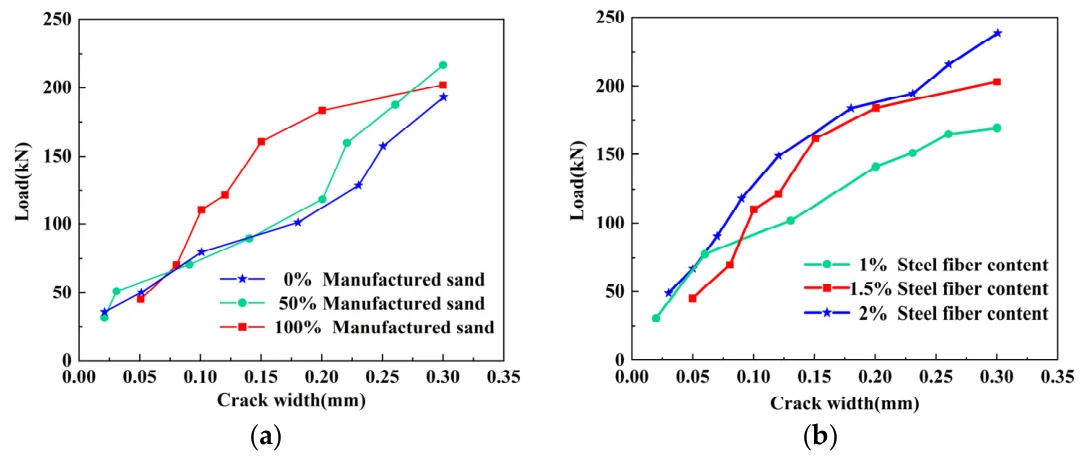
Maximum crack width: (**a**) Replacement ratios of different MS (**b**) Different steel fiber contents.

The crack comparison card was used to observe and record crack development during the experiment. Crack width observation was halted upon reaching 0.3 mm during loading. The variation oin maximum crack width in the pure bending section is illustrated in Figure 8. Following the completion of the elastic loading phase and at a load of 150 kN, the test beams ranked by decreasing main crack width, as shown in Figure 8a, are: M0-S1.5, M50-S1.5, and M100-S1.5. Notably, the test beam with 100% MS replacement ratio displayed a slower crack propagation. As illustrated in Figure 8b, the test beam containing 1% steel fiber content demonstrated accelerated crack development and wider main cracks. In contrast, the test beams with 1.5% and 2% steel fiber content exhibited similar crack development patterns. These observations suggest that enhancing the MS replacement ratio and increasing the steel fiber volume fraction in UHPMRC beams can effectively constrain the progression of crack width.

### 3.2. Load Versus Mid-Span Deflection

The experiment utilized mid-span deflection of UHPMRC beams calculated by subtracting displacements at the supports to mitigate errors arising from support settlement. The load-deflection relationships of UHPMRC beams were compared and analyzed for different influencing variables.

#### 3.2.1. Effect of MS Replacement Ratio

**Figure 9:**
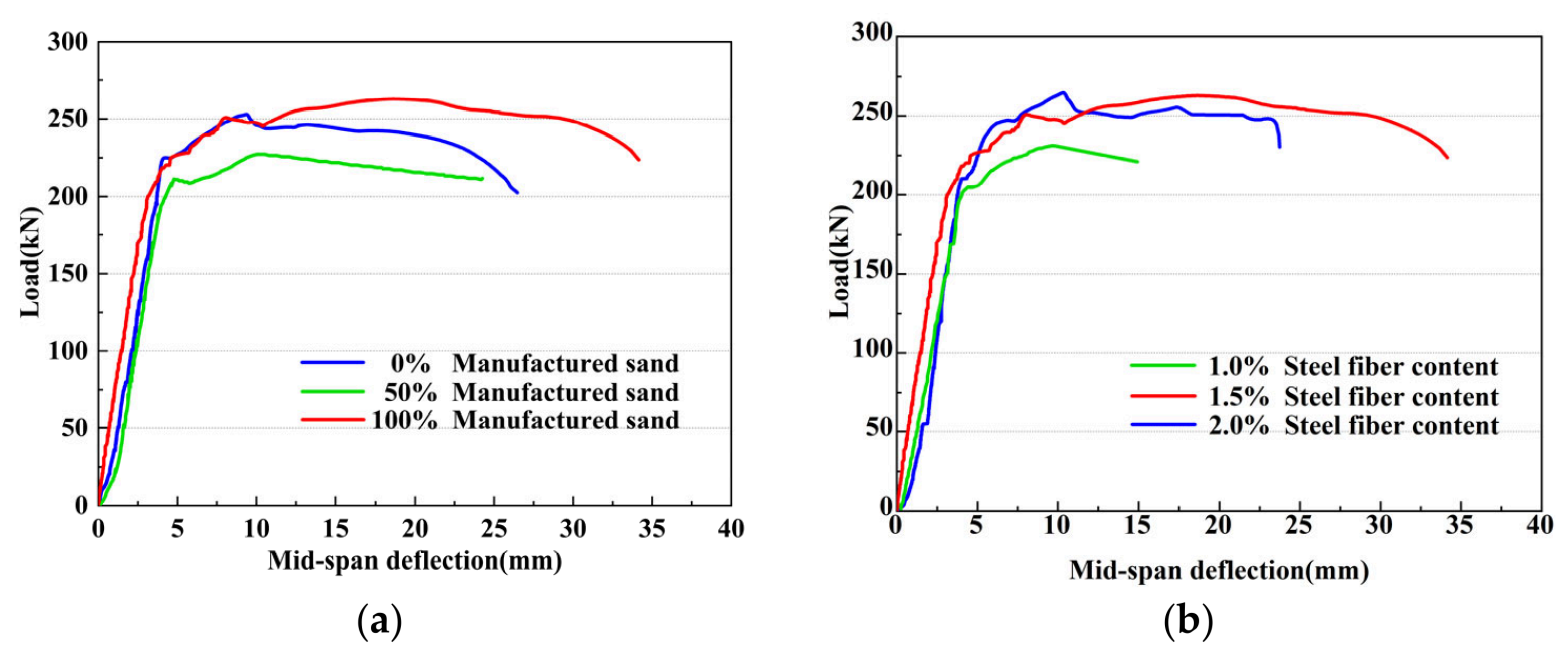
UHPMRC beam load-midspan deflection: (**a**) replacement ratios of different MS; (**b**) different steel fiber contents.

Figure 9a illustrates the mid-span load–deflection curves of UHPMRC beams under different MS replacement ratios. It can be observed that the load–deflection curves under different MS replacement ratios exhibit similar developmental trends, encompassing an elastic stage, an elastoplastic stage, and a failure stage. During the elastic stage, the load-deflection curves exhibit linear characteristics, with similar slope values observed across the three test beams. In the elastic stage, the load-deflection curve exhibits linear characteristics. From the slope of the curves of the three test beams; it can be seen that replacing quartz sand with 100% manufactured sand slightly increases the stiffness of UHPMRC beams in the elastic stage and improves the overall flexural capacity. This phenomenon can be attributed to the irregular shape of the MS, which facilitates bonding between aggregates and cement.

#### 3.2.2. Effect of Steel Fiber Content

Figure 9b illustrates the mid-span load–deflection curves of UHPMRC beams with different steel fiber contents. In the elastic stage, it is observed that the steel fiber content of UHPMRC beams with curve slope from large to small is 2.0%, 1.5% and 1.0% in order. UHPMRC beams containing 1.5% and 2.0% steel fibers exhibit significantly superior flexural capacity. This indicates that increasing the steel fiber content notably enhances the initial stiffness and load-bearing capacity of the beams. Furthermore, there is minimal difference in flexural capacity between beams with 1.5% and 2.0% fiber content. The increase in volume fraction of steel fibers within UHPMRC within a certain reasonable range results in the uniform distribution of steel fibers within the UHPMRC structure, effectively bridging between the cementitious matrix and aggregates, and enhancing its toughening effect. Consequently, UHPMRC beams exhibit higher flexural load-bearing capacity during the loading process.

#### 3.2.3. Summary of UHPMRC Beams Experimental

The mechanical metrics obtained from the experiments are shown in Table 5. In comparison to beams with 0% MS replacement ratio, those with 100% MS replacement ratio showed a 3.7% higher peak load, while beams with 50% MS replacement ratio demonstrated a 10.3% lower peak load. This indicates that the load-bearing capacity of UHPMRC beams may not increase monotonically with the MS replacement ratio. This phenomenon may be attributed to the fact that the mixed aggregate system does not necessarily form an optimized aggregate skeleton. Compared with a single-aggregate system, the combined use of MS and quartz sand may reduce the compactness of the matrix due to differences in particle morphology and packing characteristics. In addition, the nonuniform particle packing may result in a more uneven load-transfer mechanism within the matrix. The peak loads of test beams containing 1.5% and 2% steel fibers increased by 13.8% and 15%, respectively, compared to specimens with 1% steel fiber content. It is evident that increasing the steel fiber content significantly enhances the load-bearing capacity of UHPMRC beams. The cracking load of test beams with 2% steel fiber content was lower than that of samples with 1.5% content. This is primarily due to the higher fiber content of 2%, which increases the likelihood of fiber clustering during casting, resulting in poorer dispersion.

### 3.3. Load-Strain Response

#### 3.3.1. Load-Longitudinal Reinforcement Strain at Mid-Span

**Figure 10:**
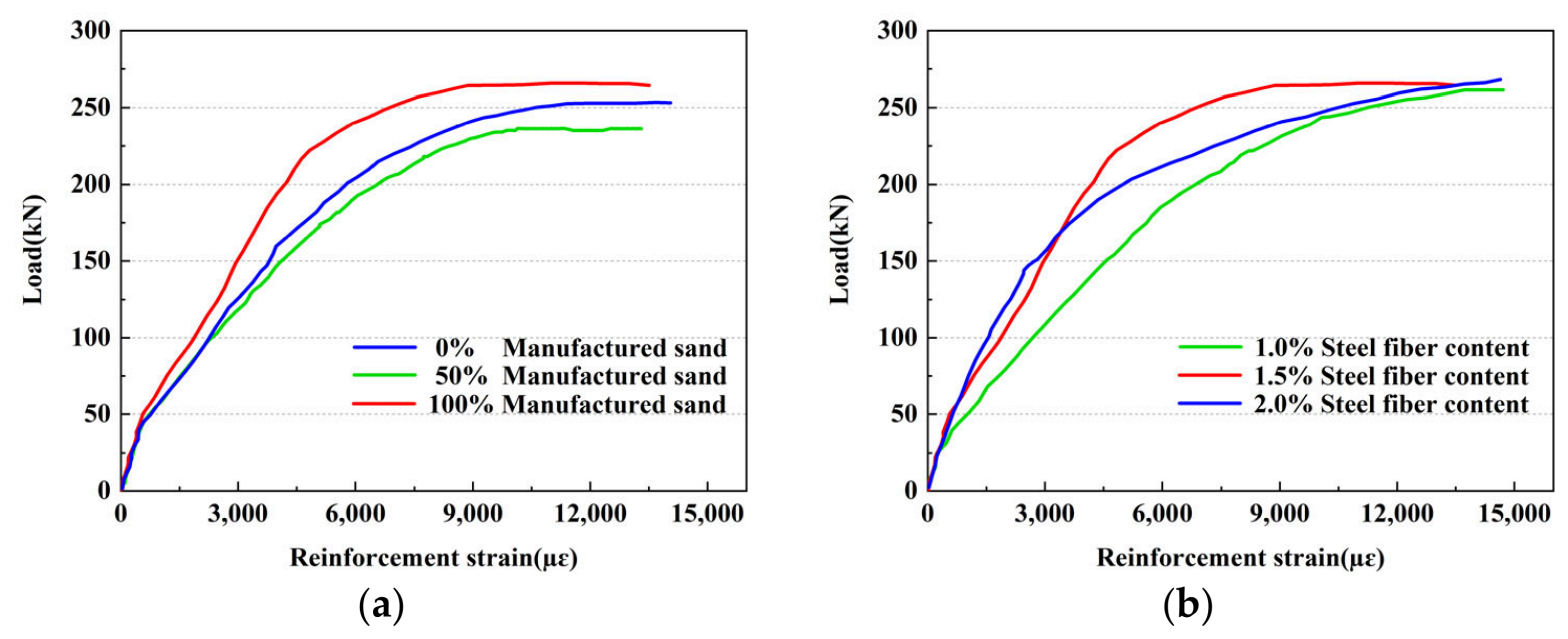
Relationship between load and reinforcement strain: (**a**) replacement ratios of different MS; (**b**) different steel fiber contents.

Figure 10 presents the strain response curve of longitudinal reinforcement at the mid-span position under varying applied loads. From the figure, it can be observed that before the test beam cracks, the load–strain curves of the reinforcement exhibit a linear relationship and are nearly coincident, indicating that the structure is in the elastic stage. After the test beam cracks, the stress in the tensile zone of the cross-section redistributes, and the tensile forces in the UHPMRC are transferred to the tensile reinforcement and the steel fibers around the cracks. As the applied load increases, the strain in the longitudinal reinforcement increases rapidly.

From Figure 10a, it is evident that under the same applied load, the strain of the reinforcement in the test beams with 100% MS replacement ratio is less than that in the beams with 0% MS replacement ratio. This phenomenon arises due to the continuous grading and unique morphology of the MS, which enhance the bond between the cement paste and the reinforcement. During the structural loading process, the test beams with 100% MS replacement ratio impose less constraint on the deformation of the bottom longitudinal bars.

From Figure 10b, it can be observed that under the same applied load, the strain of the reinforcement in the test beams with 1.5% and 2.0% steel fiber content is less than that in the beams with 1.0% steel fiber content. With the increase in load, the steel fibers in the UHPFRC cooperate with the matrix materials such as aggregates, spanning across cracks to bear a portion of the tensile stress.

#### 3.3.2. Load–Concrete Strain at Mid-Span

**Figure 11:**
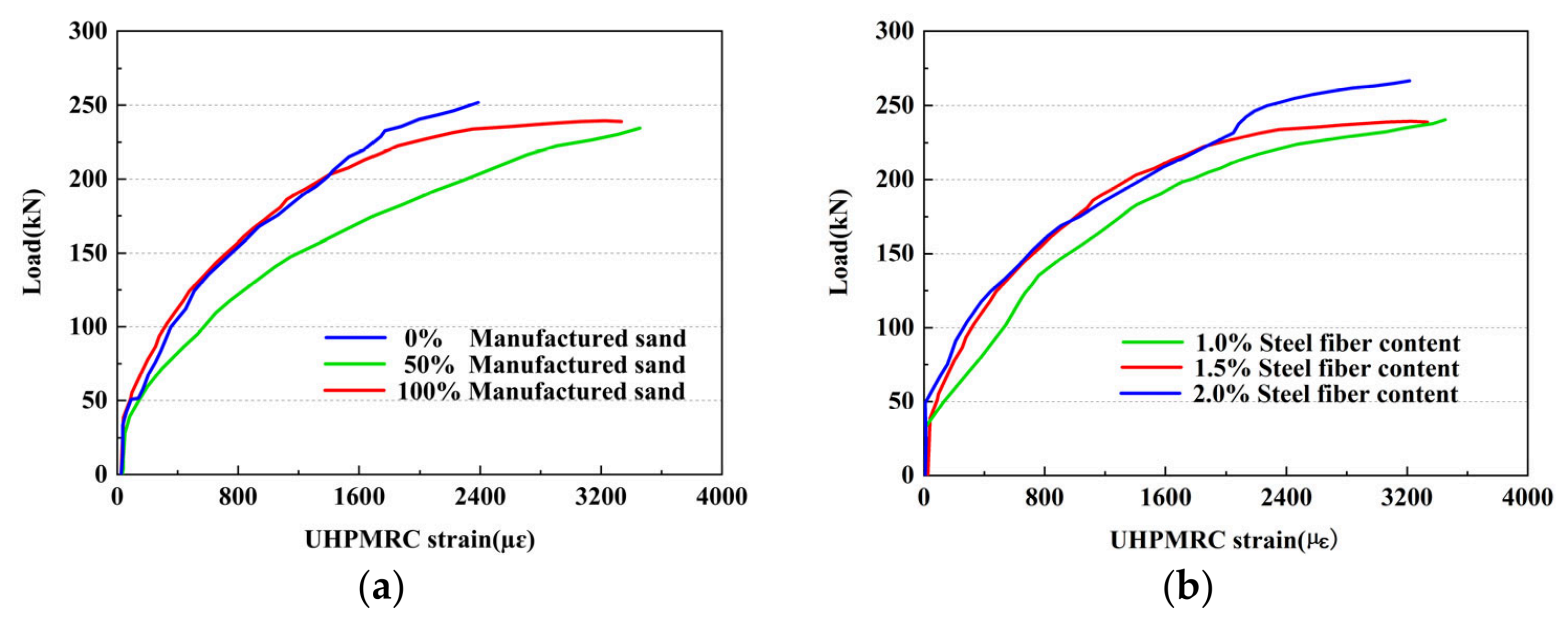
Relationship between load and UHPMRC strain: (**a**) Replacement ratios of different MS; (**b**) Different steel fiber contents.

Figure 11 shows the load–strain curve for the concrete at the bottom of the UHPMRC beam span. The figure reveals obvious inflection points in the strain behavior of the UHPMRC. Prior to cracking, the strain in the UHPMRC is very small and the mid-span cross-section stiffness is large. After the beam cracks, the strain in the tensile zone of the UHPMRC increases rapidly.

From Figure 11a, it can be observed that the overall strain development of the 100% MS replacement ratio of UHPMRC beam is generally consistent with that of the ordinary UHPRC beam. Figure 11b shows that, under the same load, the concrete strain in the test beams with 1.5% and 2.0% steel fiber content is smaller compared to the beam with 1.0% steel fiber content. Additionally, the strain curves for the beams with 1.5% and 2.0% steel fiber content are nearly coincident. This is because with the increase in volume fraction of steel fibers, the bridging effect of steel fibers within the cracks of the beam also intensifies. However, excessive steel fiber content may lead to clustering, insufficient bonding with the cementitious matrix, and thereby limit the enhancement effect.

#### 3.3.3. Distribution of Longitudinal Strain Along the Height of Cross Section

**Figure 12:**
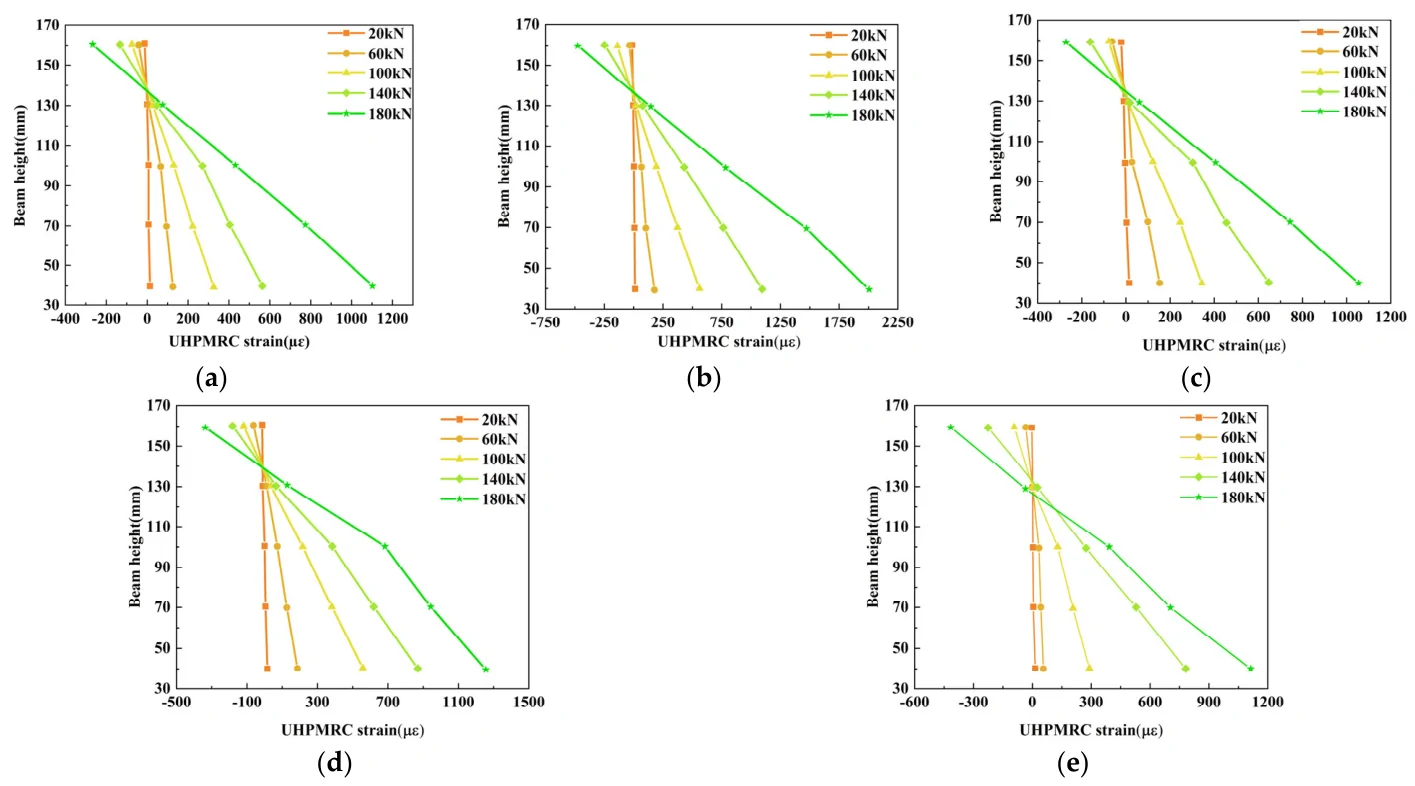
UHPMRC mid-span cross-section strain: (**a**) M100-S1.5; (**b**) M50-S1.5; (**c**) M0-S1.5; (**d**) M100-S1.0; (**e**) M100-S2.0.

Figure 12 illustrates the strain variation of the mid-span section of the UHPMRC beam under different load levels. It can be observed that before cracking, the strain distribution at the mid-span cross-section is essentially linear. After cracking, the stress transfers from the concrete to the reinforcement, causing the neutral axis of the mid-span cross-section to gradually shift upward. Simultaneously, with the increase in steel fiber content, the height of the neutral axis decreases. The bonding between fibers and the matrix enhances the tensile force provided by the concrete in the tension zone and increases the height of the compressive zone. Throughout the loading process, the strain distribution at mid-span of each test beam generally exhibits a linear pattern along the cross-sectional height, conforming to the assumption of flat cross-section. It is noteworthy that specimen M50-S1.5 exhibits a greater variation in strain compared to the other specimens. This is attributed to the fact that at a MS content of 50%, the coexistence of rough MS and smooth quartz sand maximizes the difference in mechanical properties of the interfacial transition zone, leading to the most significant microscopic stress concentration, which macroscopically manifests as the largest variation in longitudinal strain distribution along the cross-sectional height.

### 3.4. Ductility and Flexural Toughness

**Figure 13:**
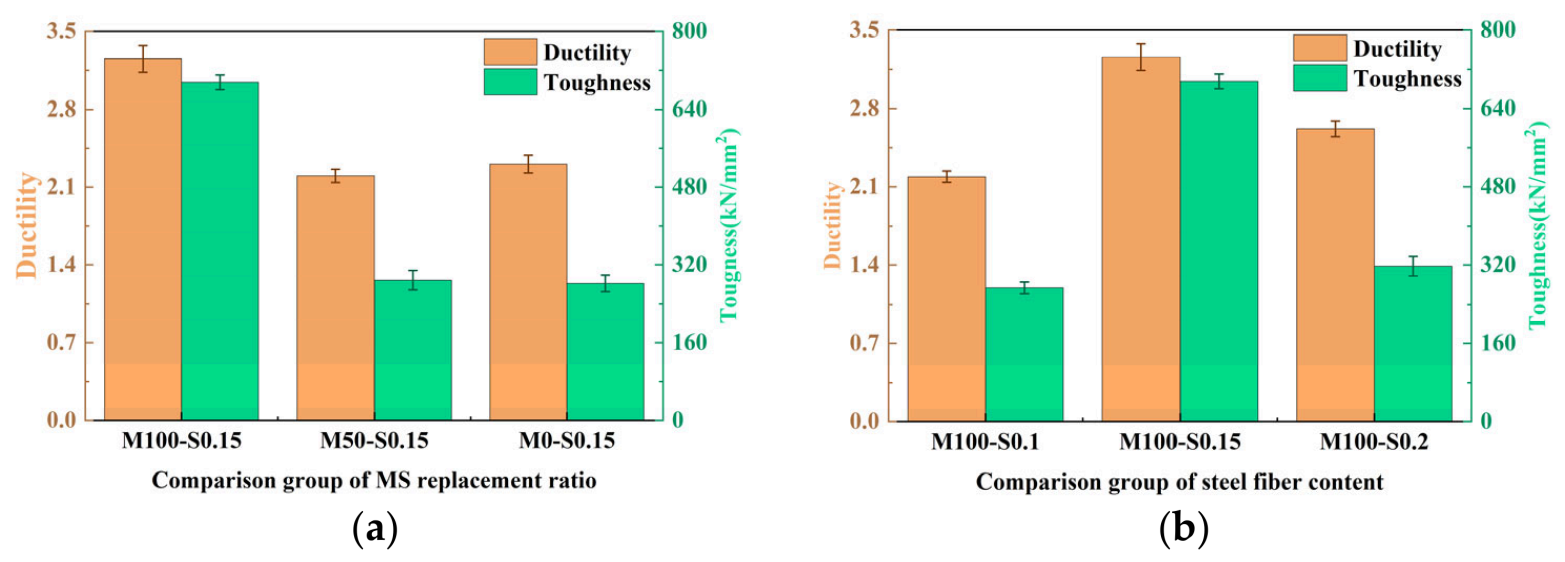
Comparison of ductility and flexural toughness: (**a**) Replacement ratios of different MS; (**b**) different steel fiber contents.

According to Table 5, the ductility indices of the tested UHPMRC beams are all greater than 2, indicating that these beams effectively utilize the strain hardening characteristics of UHPMC after reinforcement yielding. This satisfies the ductility requirements for flexural members, demonstrating good ductility performance. From Figure 13, it can be observed that the MS replacement ratios positively influences the ductility and flexural toughness of UHPMC beams. The effect of steel fibers on the ductility of the test beams is more pronounced. Compared to the sample with 1.0% steel fiber content, the ductility index of the beams with 1.5% steel fibers increased by 49%, and the flexural toughness increased by 119%. However, compared to the sample with 1.5% steel fibers, the ductility and flexural toughness of the beams decreased with a steel fiber content of 2.0%. It is evident that the phenomenon of steel fiber clustering significantly affects the ductility and flexural toughness of the test beams. Among these, the beams with 100% MS replacement ratio and 1.5% steel fiber content exhibit the best ductility and flexural toughness performance. The ductility is 41% higher compared to the 0% MS sample, and the flexural toughness is 134% higher compared to the 0% MS sample.

### 3.5. Energy Absorption Capacity

The energy absorption value was obtained by integrating the load–deflection curve using Origin software (Version 2022). The slight enhancement in energy absorption is attributed to rapid concrete beam failure under relatively high loads. The substantial increase in energy absorption is associated with increased post-yield loads and enhanced stiffness. According to Table 5, compared to the beams with 0% MS replacement ratio, those with 50% and 100% replacement show improvements. Similarly, compared to beams with 1.0% steel fiber content, those with 1.5% and 2.0% also demonstrate enhancements. The test beams with a 100% MS replacement ratio and 1.5% steel fiber content exhibit a significant increase in energy absorption values, indicating superior overall performance.

### 3.6. Stiffness Degradation Analysis

**Figure 14:**
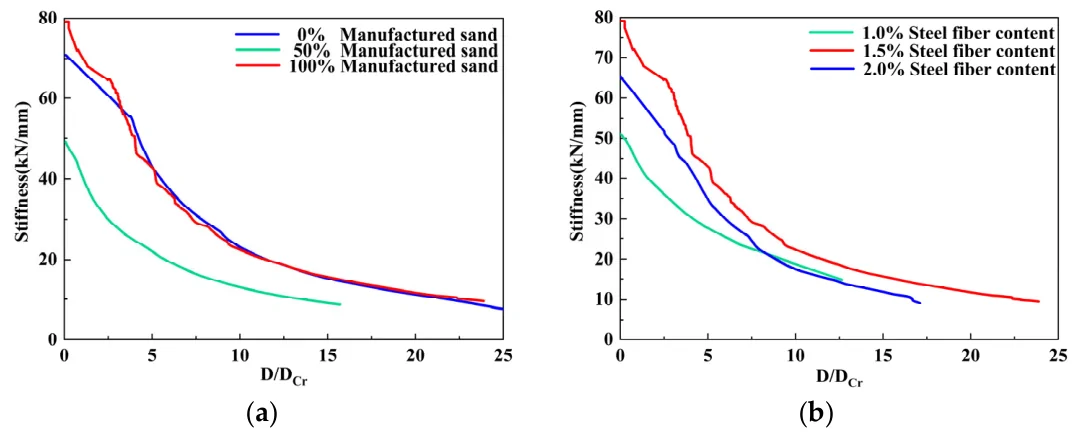
Stiffness degradation analysis: (**a**) Replacement ratios of different MS; (**b**) different steel fiber contents.

Figure 14 illustrates the stiffness degradation results of UHPMRC beams. The horizontal axis represents the ratio of the mid-span deflection (*Δ*) to the mid-span deflection (*Δcr*) at flexural cracking; the vertical axis represents the stiffness calculated from the secant slope of the load–deflection curves. The stiffness degradation trends of the test beams exhibit significant variation under different parameters.

#### 3.6.1. Effect of MS Replacement Ratio

As shown in Figure 14a, MS significantly influences the initial stiffness of UHPMRC beams. Among these, the beam with 50% MS replacement ratio exhibits the lowest initial stiffness and the fastest rate of stiffness degradation. The beam with 100% MS replacement ratio shows slightly better initial stiffness compared to the conventional UHPC beam, with a slightly faster degradation rate. This suggests that replacing QS entirely with MS (100% replacement) in preparing UHPMRC beams does not significantly affect their stiffness.

#### 3.6.2. Effect of Steel Fiber Content

Figure 14b indicates that increasing the steel fiber content positively affects the initial stiffness of UHPMRC beams. Comparing the stiffness degradation curves of the three beams, the beam with 1.5% fiber content demonstrates superior initial stiffness and exhibits better stiffness retention after cracking.

## 4. Fracture Performance of Reinforced UHPMRC

### 4.1. AE Energy and Cumulative Energy

AE energy refers to the elastic energy released by sources of AE, such as internal damage within the test beam or cracking in UHPMC. It represents the residual elastic energy detected on the surface of the test beam after attenuation during the propagation of AE. The AE energy is primarily divided into three stages:

- Linear elastic deformation: At this stage, the AE energy is minimal. Since no cracks form within the specimen during this phase, the AE energy remains at a relatively low level. The cumulative AE energy also exhibits a nearly parallel trend with the *X*-axis.
- Stable crack propagation: At this stage, the AE energy begins to exhibit relatively high values. Cracks develop within the specimen, and as these cracks propagate, the cumulative AE energy increases to varying degrees. The signals observed during this phase primarily originate from cracking within the concrete matrix and the pull-out of steel fibers.
- Unstable crack propagation: During this stage, the AE energy reaches its maximum value. The cumulative energy growth rate achieves its peak. At this point, macro-cracks in the specimen open up and interconnect. The AE signals primarily originate from concrete matrix cracking, steel fiber pull-out, and reinforcement deformation.

#### 4.1.1. Effect of MS Replacement Ratio

**Figure 15:**
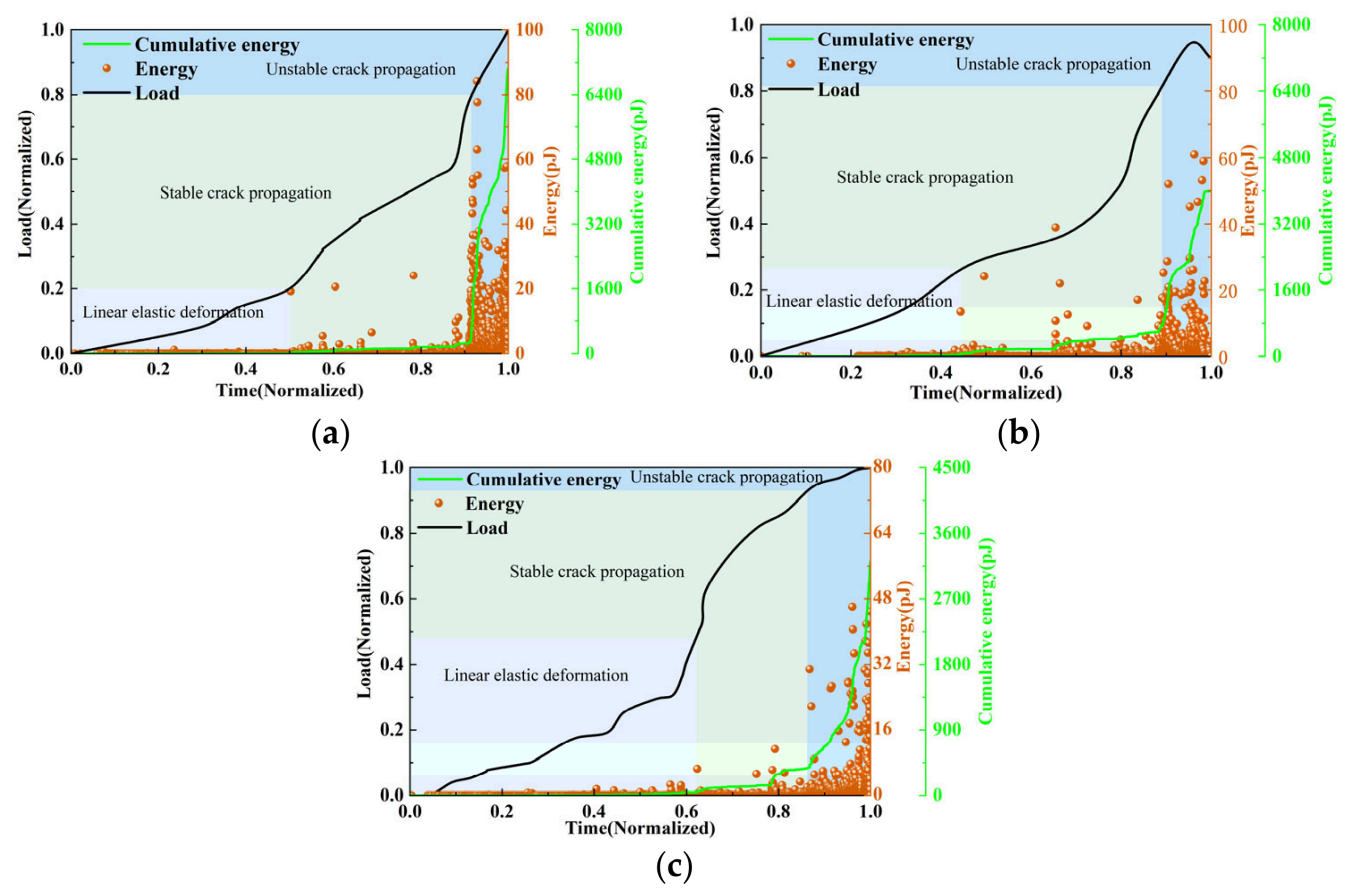
UHPMRC beam AE energy and cumulative energy: (**a**) 0% MS; (**b**) 50% MS; (**c**) 100% MS.

Figure 15 illustrates the AE energy of beams with varying MS replacement ratio. The load level corresponding to the end of the linear elastic deformation stage increases with the rise in MS content. For UHPMRC beams with 0%, 50%, and 100% MS content, the load levels corresponding to the end of the first stage are 0.2, 0.25, and 0.47, respectively. In other words, an increase in the content of MS leads to a higher load required for cracks to appear. This is because the rough surface and angular characteristics of MS enhance the mechanical interlocking force between the sand and the matrix, thereby improving the resistance to cracking. In the stable crack propagation stage, some high energy appeared. Compared to the 0% and 50% MS UHPMRC beams, the energy at 100% MS was relatively low. According to [46,47], the energy released by AE events is generally proportional to the size of cracks. This indicates minimal internal damage to the beam. In the unstable crack propagation stage, cracks interconnect, and the energy value reaches its maximum. The maximum energy value at 0% is 85 pJ, at 50% is 62 pJ, and at 100% is 48 pJ. As the content of MS increases, both the maximum energy value and cumulative energy decrease. This indicates that as the content of MS increases, the damage level of UHPMRC beams decreases. For the 50% MS specimens, the enhanced interfacial bonding force due to MS transforms the crack propagation mode from fewer but larger cracks to more but smaller micro-cracks. Although this more distributed micro-crack damage pattern macroscopically manifests as larger strain, it effectively inhibits the formation of penetrating large cracks; consequently, the internal damage degree (AE energy) is conversely lower, reflecting superior toughness.

#### 4.1.2. Effect of Steel Fiber Content

**Figure 16:**
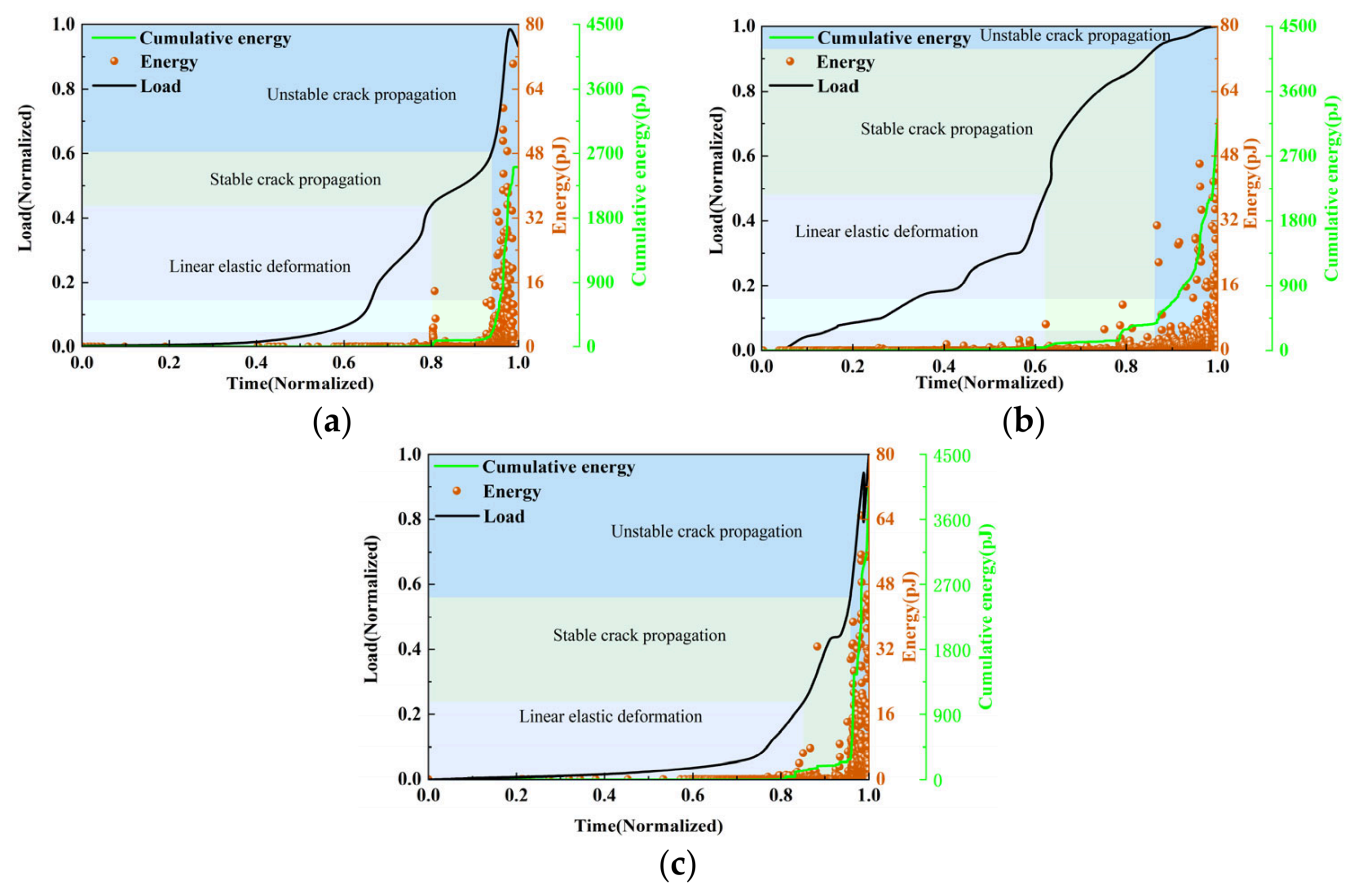
UHPMRC beam AE energy and cumulative energy: (**a**) 1.0% steel fiber; (**b**) 1.5% steel fiber; (**c**) 2.0% steel fiber.

The AE energy of test beams with varying steel fiber contents is depicted in Figure 16. Compared to specimens with 1.0% and 2.0% steel fiber content, specimens with 1.5% steel fiber exhibited a higher load level at the end of the linear elastic stage. Steel fibers were uniformly distributed in specimens with both 1.0% and 1.5% steel fiber content. Due to the higher steel fiber content per unit area in the 1.5% specimens, the bridging effect of the steel fibers is enhanced, resulting in a higher load at which cracks appear. Specimens containing 2.0% steel fibers fail to achieve their intended reinforcement effect due to excessive fiber agglomeration. This tendency to form weak bond interfaces between steel fibers and the matrix reduces the bridging effect, leading to earlier crack initiation. In the stable crack propagation stage, the steel fiber distribution, demonstrating high toughness during the stable crack propagation phase. The energy absorbed by the 1.5% specimens was comparatively lower than the other two specimens, indicating the most gradual crack development. These specimens also exhibited a longer stable crack propagation phase. The third stage represents the phase of unstable crack propagation. The maximum energy values for specimens with 1.0%, 1.5%, and 2.0% steel fiber content were 70 pJ, 47 pJ, and 65 pJ, respectively. During final failure, the 1.5% steel fiber specimen exhibited a more gradual failure mechanism, attributed to the full utilization of the steel fibers bridging effect. The energy distribution observed in the 1.5% steel fiber specimen was relatively dispersed, further indicating a more dispersed crack propagation pattern. The cumulative energy increases with the steel fiber content. This is because a higher steel fiber content involves more steel fibers in the drawing process, thereby generating more friction signals and ultimately resulting in the maximum cumulative energy value.

### 4.2. Correlation Between AE Energy and Energy Absorption Capacity

**Figure 17:**
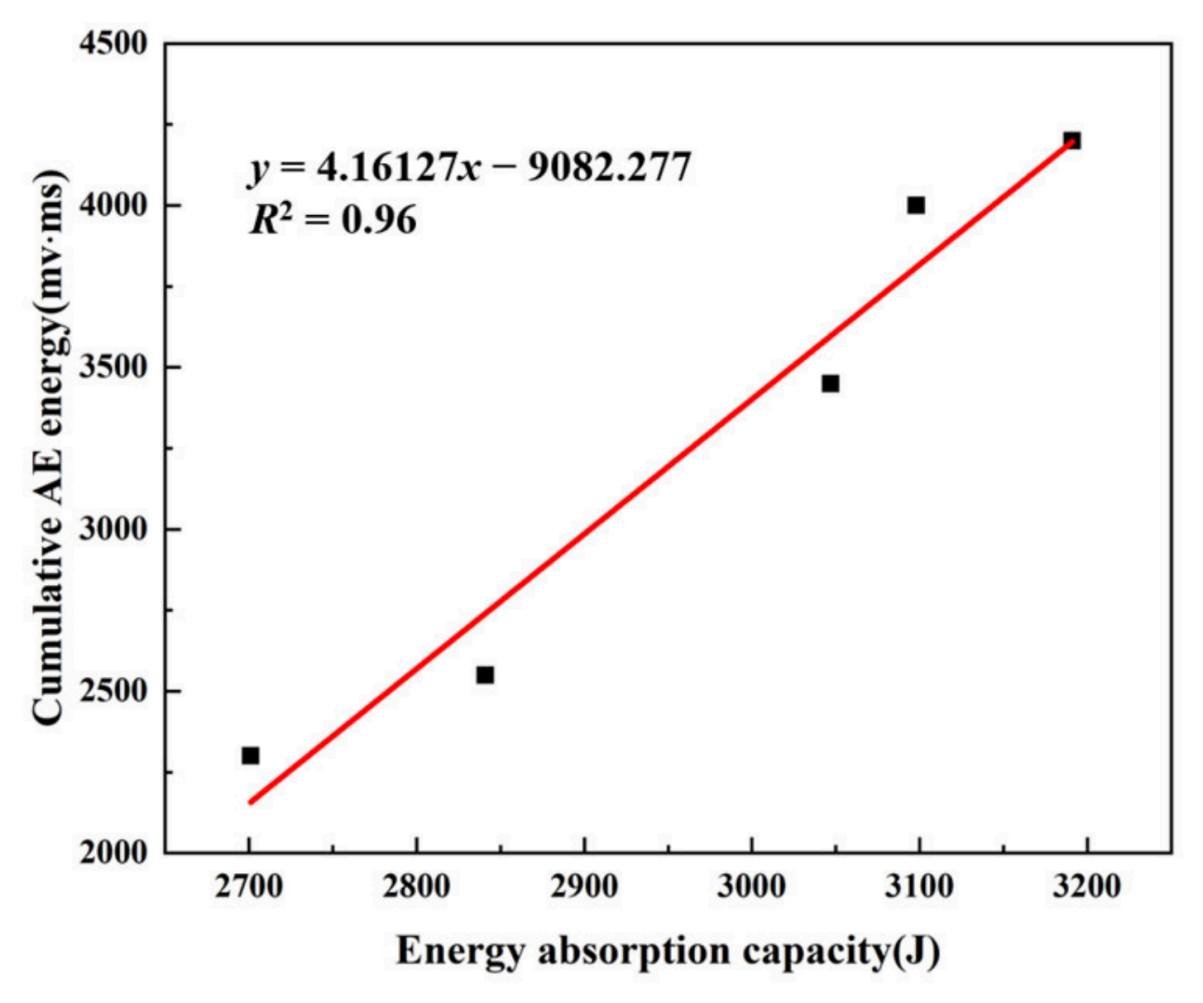
Cumulative AE energy as a function of energy absorption.

Cumulative AE energy correlates with energy absorption values [34]. Under external loading, the work done by the external force is stored internally as strain energy within the material. When the locally stored strain energy exceeds the structural capacity to withstand local strains, localized damage occurs in the structure, releasing energy. The relationship between cumulative AE energy and energy absorption values is depicted in Figure 17. As the energy absorption capacity of UHPMRC beams increases, there is a corresponding upward trend in cumulative AE energy. Cumulative energy and energy absorption values are two distinct physical concepts, yet they exhibit a consistent qualitative relationship in the flexural tests of UHPMRC beams. This qualitative relationship aligns with the conclusions drawn in the literature regarding reinforced concrete structures [48,49,50].

### 4.3. b-Value

#### 4.3.1. Effect of MS Replacement Ratio

**Figure 18:**
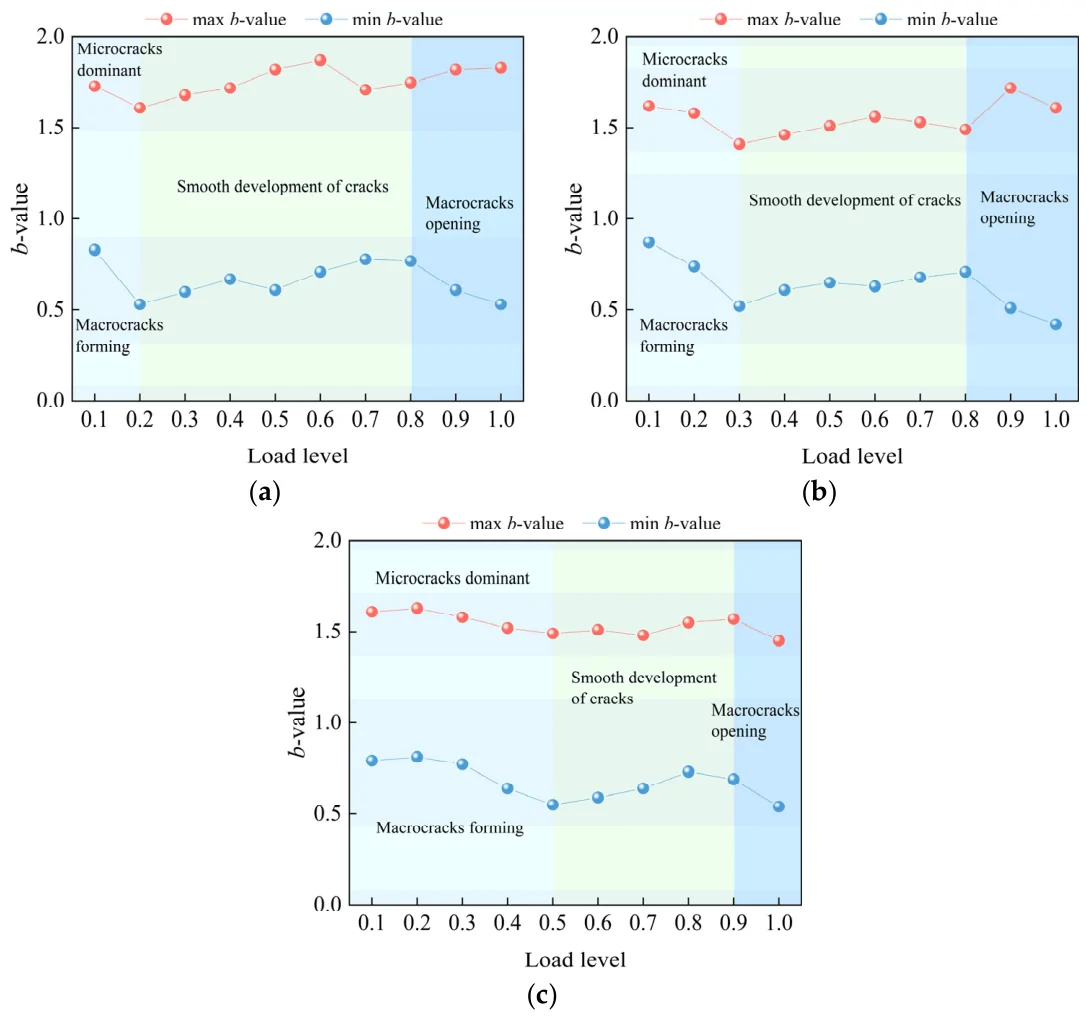
AE *b*-values of UHPMRC beams with different MS replacement ratios: (**a**) 0% MS; (**b**) 50% MS; (**c**) 100% MS.

The *b*-values of UHPMRC beams with varying MS content are presented in Figure 18. The maximum *b*-value for 0% MS exhibits a noticeable decline at the 0.2 load level, indicating microcrack formation. The minimum *b*-value shows a sudden drop at 0.2, signifying the emergence of macrocracks. Visible cracks were also observed during testing. Load levels 0.2–0.8 exhibit relatively stable behavior, with macro-cracks developing steadily during this phase. At load levels 0.8–1.0, the *b*-value decreases as macrocracks open to form the maximum principal crack, leading to specimen failure. The minimum *b*-value for 50% MS exhibits a noticeable change at 0.3, indicating the emergence of microcracks. A decrease in the minimum *b*-value at 0.3 signifies the appearance of macrocracks. A further decline occurs at the 0.8–1.0 load level, corresponding to the opening of macrocracks. The maximum *b*-value for 100% MS decreases at 0.5, indicating the emergence of microcracks. The minimum *b*-value decreases at the 0.5 load level, signifying the appearance of macrocracks. A further decrease occurs at the 0.9 load level, where interconnected macro-cracks form the primary crack network. As shown in the figure, with increasing content of MS, the load level at which macroscopic cracks appear becomes progressively higher. The load level at which the ultimate main crack forms is also higher.

#### 4.3.2. Effect of Steel Fiber Content

**Figure 19:**
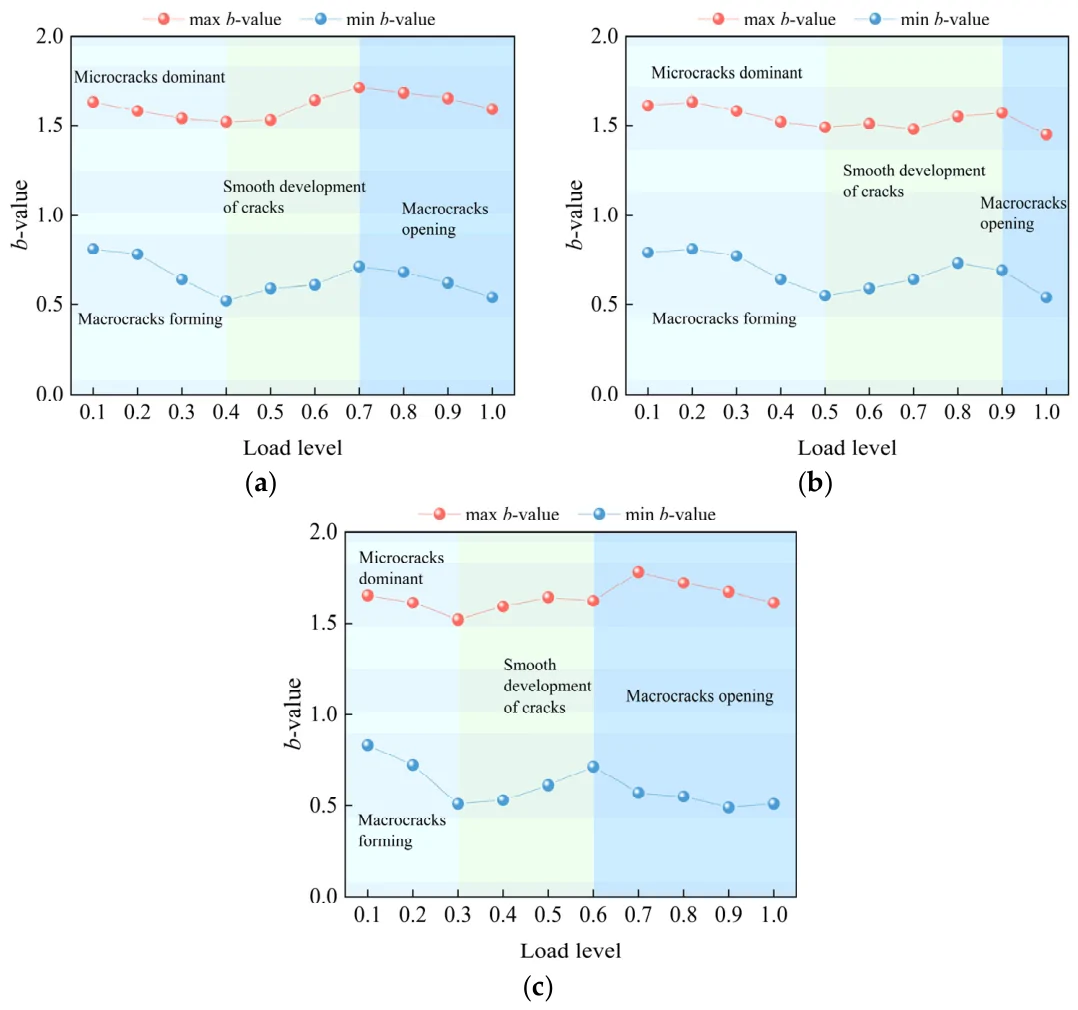
AE *b*-values of UHPMRC beams with different Steel fiber contents: (**a**) 1.0% steel fiber; (**b**) 1.5% steel fiber; (**c**) 2.0% steel fiber.

The *b*-values of UHPMRC beams with different steel fiber contents are illustrated in Figure 19. The maximum *b*-value for 1.0% steel fiber content decreased at load level 0.4, indicating microcrack formation. The minimum *b*-value decreased at load level 0.4, signifying macrocrack formation. A decrease occurred at load level 0.7, at which point macrocracks began to open and form primary cracks. At a steel fiber content of 1.5%, the maximum *b*-value decreased at 0.5, indicating microcrack formation. At the 0.9 load level, another abrupt change occurred, with macrocracks opening to form primary cracks. The maximum *b*-value for 2.0% steel fiber reinforcement decreased at the 0.3 load level, with microcracks becoming dominant. At the 0.3 load level, the maximum *b*-value decreased as macrocracks had already formed. At the 0.6 load level, the minimum *b*-value decreased as macrocracks began to develop into primary cracks. Compared to specimens with 1.0% and 2.0% steel fiber content, specimens with 1.5% steel fiber exhibited macroscopic cracking at higher load levels. This occurs because an optimal amount of steel fiber provides the best bridging effect, more effectively inhibiting crack propagation. Specimens with either insufficient or excessive steel fiber content showed macroscopic cracks developing earlier and progressing toward primary cracks.

### 4.4. Crack Classification Based on RA-AF

#### 4.4.1. Effect of MS Replacement Ratio

**Figure 20:**
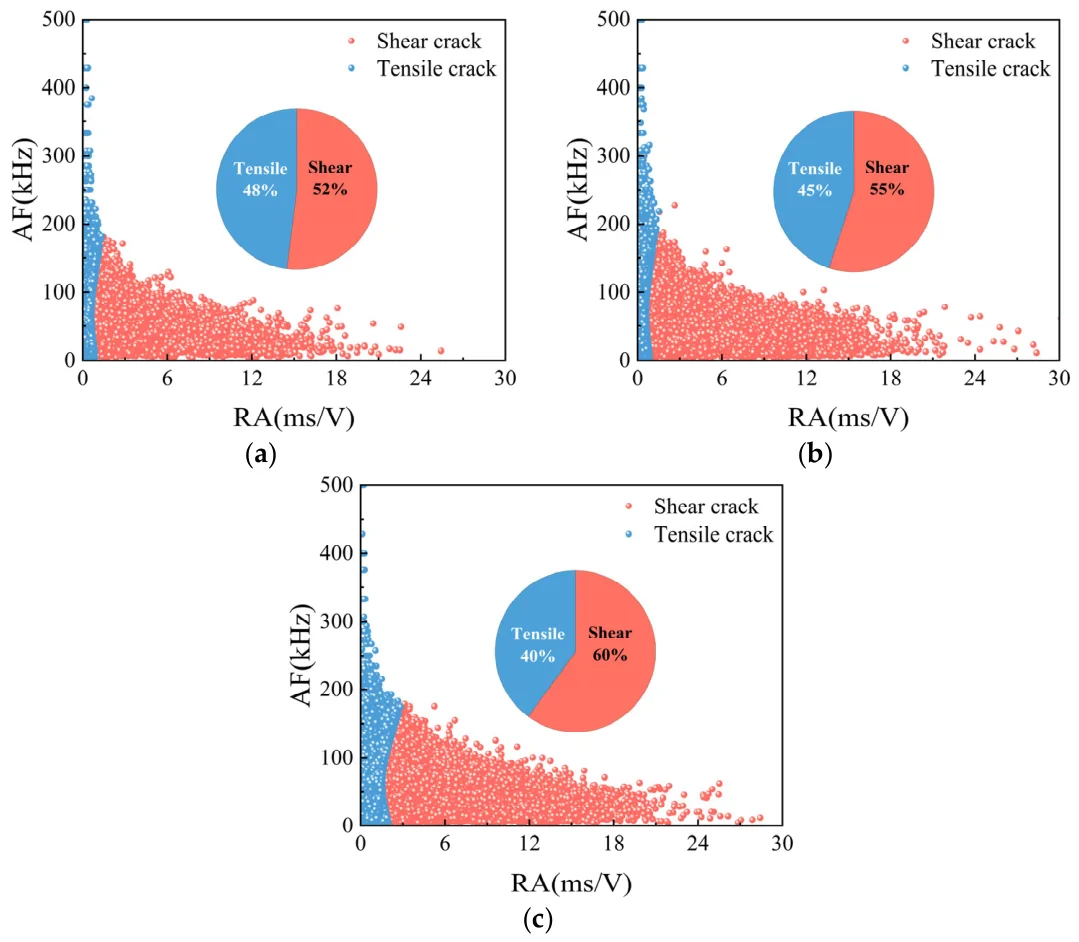
RA-AF of UHPMRC beams with different MS replacement ratios: (**a**) 0% MS; (**b**) 50% MS; (**c**) 100% MS.

The RA-AF of UHPMRC beams with different MS contents are presented in Figure 20. UHPMRC beams with 0%, 50%, and 100% MS content exhibited shear crack lengths of 52%, 55%, and 60%, respectively, during fracture. The fracture process of all UHPMRC beams was dominated by shear signals. As the content of MS increases, the shear signals generated during specimen failure become more pronounced while tensile signals diminish. The matrix develops tensile cracks under tensile stress, and the angular characteristics of MS enhance the mechanical interlocking force between matrix particles, thereby inhibiting the propagation of tensile cracks within the matrix.

#### 4.4.2. Effect of Steel Fiber Content

**Figure 21:**
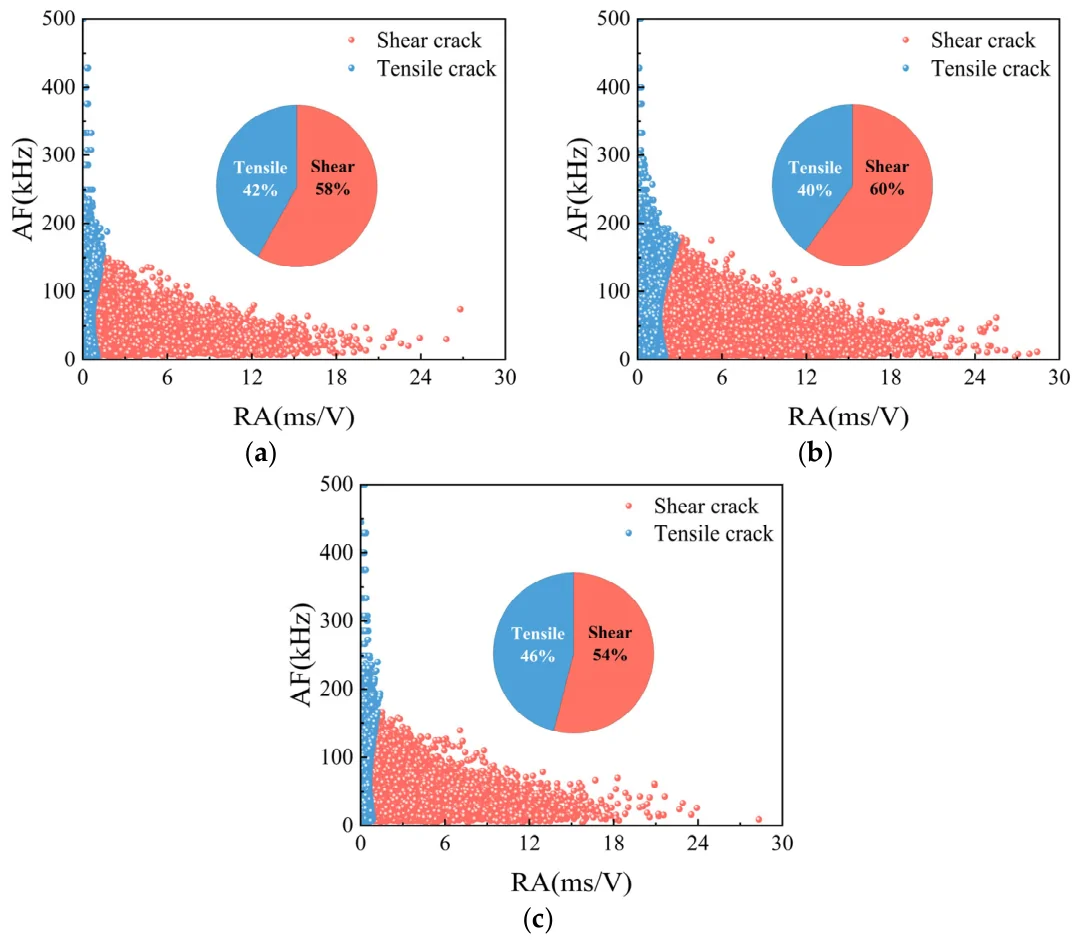
RA-AF of UHPMRC beams with different steel fiber contents: (**a**) 1.0% steel fiber; (**b**) 1.5% steel fiber; (**c**) 2.0% steel fiber.

The RA-AF of UHPMRC beams with different steel fiber contents is illustrated in Figure 21. UHPMRC beams with steel fiber contents of 1.0%, 1.5%, and 2.0% exhibited shear crack proportions of 58%, 60%, and 54%, respectively, during fracture. The signal generated during steel fiber pull-out is a shear signal. When the steel fiber content increases from 1.0% to 1.5%, the proportion of shear signal increases. One reason is that in specimens with 1.5% steel fiber content, the fracture surface exhibits more uniform and denser steel fiber distribution, providing better resistance to tensile cracking. Another reason is that a greater number of steel fibers participate in the pull-out process, consequently increasing the shear signal. The steel fiber content was increased from 1.5% to 2.0%, resulting in a decrease in the shear signal ratio. This is because excessively high steel fiber content causes agglomeration of steel fibers and the formation of steel fiber voids, thereby weakening the material ability to resist tensile cracking. Consequently, the shear signal ratio diminishes.

## 5. Discussion

This study systematically investigated the flexural and fracture behaviors of UHPMRC beams through four-point bending tests coupled with AE monitoring. Compared with the existing literature, the novelty and originality of this work are threefold.

First, regarding the effect of MS replacement ratio, previous studies were largely confined to material-level specimens. Donza et al. [16] reported that the angular morphology of MS enhances interlocking between particles, Yang et al. [17] found that 80% substitution of quartz sand yielded compressive strength exceeding that of conventional UHPC mixes, and Su et al. [18] observed better flexural capacity at a 50% replacement rate in plain UHPMC beams. In contrast, this study demonstrates for the first time at the reinforced beam component level that 100% MS substitution is not only feasible but advantageous: compared with 0% MS specimens, the peak load increased by 3.7%, while ductility and flexural toughness improved by 41% and 134%, respectively. This discrepancy arises from the synergistic action of steel rebars and steel fibers—the rough surface of MS enhances aggregate–paste interfacial bonding, reducing localized strain concentrations (Figure 10a) and allowing the reinforcement to participate more efficiently. Furthermore, *b*-value analysis quantitatively reveals that increasing MS content raises the load level for macrocrack initiation from 0.2 (0% MS) to 0.5 (100% MS), a crack-inhibiting effect not previously quantified in material-level tests.

Second, concerning steel fiber content, the 1.5% dosage yielded optimal ductility, toughness, and crack control, whereas the 2.0% dosage led to fiber clustering, resulting in a lower cracking load and reduced ductility and toughness. While this trend generally agrees with Hasgul et al. [22] regarding fiber contributions to flexural capacity, our work further identifies a clear threshold effect—at 2.0% fibers, agglomeration creates weak interfaces, and RA-AF analysis shows that the shear-signal proportion drops from 60% (1.5%) to 54% (2.0%), indicating that fewer fibers effectively participate in pull-out. This provides component-level validation that 1.5% is the recommended fiber content, challenging the simplistic notion that “more fibers always improve performance”.

Third, in terms of AE methodology, this study integrates AE energy, *b*-value, and RA-AF analyses to characterize the entire damage process of UHPMRC beams. The three-stage evolution of AE energy aligns with findings for conventional concrete [34,36], but the elastic stage in UHPMRC beams extends to 0.47 of the ultimate load (100% MS)—markedly longer than in ordinary concrete—reflecting the combined toughening effects of MS and fibers. The qualitative correlation between cumulative AE energy and energy absorption capacity (Figure 17) is validated for the first time in MS-based UHPC, suggesting AE parameters as a non-destructive indicator of energy dissipation. RA-AF classification further quantifies that MS enrichment increases the proportion of shear cracks (52% → 60%), while fiber clustering weakens this effect.

In summary, 100% MS replacement combined with 1.5% steel fibers produces reinforced beams with flexural performance superior to conventional quartz sand UHPC, and AE monitoring effectively tracks their damage evolution. This study provides component-level scientific evidence for full MS substitution in UHPC, promoting low-cost and low-carbon applications. Future work should examine different reinforcement ratios, long-term durability, and full-scale member validation.

## 6. Conclusions

The utilization of MS in UHPMRC beams represents a pioneering study with significant benefits for the concrete industry. This approach is particularly advantageous for resource-conserving and environmentally sustainable practices, especially in developing countries. This study, based on four-point bending tests, employs AE techniques to analyze the flexural performance of UHPMRC beams from multiple perspectives. The following conclusions have been drawn:

- All UHPMRC beams exhibited flexural failure; however, the ultimate failure mode varied slightly with the replacement ratio of MS. In specimens with full replacement by manufactured sand, failure was characterized by fracture of the bottom longitudinal tensile reinforcement, indicating a tensile failure. In specimens without MS and with a 50% replacement ratio, failure was manifested by spalling of concrete in the top compression zone, while the tensile reinforcement remained unbroken, indicating a compressive failure.
- At the same MS replacement rate, increasing the steel fiber content helps to enhance crack control in UHPMRC beams and improve their flexural load-carrying capacity. Compared to a steel fiber content of 1%, the peak load increased by 13.8% and 15% for steel fiber contents of 1.5% and 2.0%, respectively. Specimens with a steel fiber content of 2% exhibited fiber agglomeration around the reinforcing bars, resulting in lower ductility and stiffness compared to specimens with a steel fiber content of 1.5%.
- Under the same steel fiber content, continuous grading MS helps achieve a more uniform stress distribution in UHPMRC. The bending performance of UHPMRC beams made with 100% MS exceeded that of beams made with 0% MS. Compared to specimens made with 0% MS, the peak load of specimens made with 100% MS increased by 3.7%. Ductility and bending toughness increased by 41% and 134%, respectively.
- Through the *b*-value analysis, it was found that as the content of MS increases, the corresponding load level at which macroscopic cracks appear becomes higher. The load level at which the ultimate main crack forms is also higher. For UHPMRC beams with a 1.0% steel fiber content, macroscopic cracks appear later during the fracture process. RA-AF analysis revealed that as the content of MS increased, the shear signals generated during specimen failure increased while tensile signals decreased. UHPMRC beams with 1.0% steel fiber content exhibited the lowest proportion of tensile cracks during fracture.

This study demonstrates, for the first time at the component level, the enhancement effect of full replacement of quartz sand by manufactured sand (MS) on the flexural behavior of UHPMRC beams and reveals that the underlying micro mechanism lies in the optimization of aggregate-paste bonding by the rough MS surface and the multi-scale synergy with steel rebars and fibers. The threshold effect of steel fiber content is identified (1.5% being optimal, while 2.0% causes performance degradation due to clustering), providing a new theoretical basis for the material–structure integrated design of ultra-high-performance concrete. From an engineering perspective, it is recommended to adopt 100% MS substitution for quartz sand combined with 1.5% steel fibers in flexural members, which offers both performance enhancement and the advantages of low carbon footprint and cost-effectiveness.

## Figures and Tables

**Table 1:** Specimen design parameters.

Specimens	MS Replacement Ratio M (%)	Steel Fiber Content S (%)
M100-S1.5	100	1.5
M50-S1.5	50	1.5
M0-S1.5	0	1.5
M100-S1.0	100	1.0
M100-S2.0	100	2.0

Notes: M”X”-S”Y” denotes reinforced UHPMC beams with MS replacement rate X% (X = 0, 50 and 100) and steel fiber content Y% (Y = 1.0, 1.5 and 2.0).

**Table 2:** UHPMRC beam mix ratio (kg/m^3^).

Specimen	Cement	SF	QS	MS	QP	Steel Fibers	Water	HPWRA
M100-S1.5	850	195.5	-	935	331.5	117.75	167.28	42.5
M50-S1.5	850	195.5	467.5	467.5	331.5	117.75	167.28	42.5
M0-S1.5	850	195.5	935	-	331.5	117.75	167.28	42.5
M100-S1.0	850	195.5	-	935	331.5	78.5	167.28	42.5
M100-S2.0	850	195.5	-	935	331.5	157	167.28	42.5

Notes: Silica fume is abbreviated as “SF”, quartz sand is abbreviated as “QS”, manufactured sand is abbreviated as “MS”, quartz powder is abbreviated as “QP”.

**Table 3:** Mechanical properties of UHPMC materials.

Specimen	M100-S1.5	M50-S1.5	M0-S1.5	M100-S1.0	M100-S2.0
*f*_cu_/MPa	131.6	124.6	127.9	120	136.8

**Table 4:** Material properties of rebar.

Grade of Steel	Diameter (mm)	Yield Strength (Mpa)	Ultimate Strength (Mpa)
HRB400	14	428.87	568.36
HRB400	6	432.65	608.44

**Table 5:** Summary of UHPMRC beams experimental results.

Specimen	Cracking		Yield		Peak		Ductility	Toughness	Energy Absorption Capacity
	*P_cr_*/kN	*Δ_cr_*/mm	*P_y_*/kN	*δ_y_*/mm	*P_m_*/kN	*δ_m_*/mm	*µ*	*E_m_*/(kN/mm^2^)	$\Omega_{m}$/(kN·mm)
M100-S1.5	55.80	0.69	228.39	5.72	263.01	18.67	3.26	695.34	4172.01
M50-S1.5	30.98	1.33	211.25	4.78	227.25	10.51	2.20	288.60	1731.60
M0-S1.5	36.07	1.02	224.41	4.09	253.39	9.47	2.31	281.94	1691.65
M100-S1.0	32.89	0.87	204.6	4.71	231.15	9.98	2.19	273.04	1638.24
M100-S2.0	39.53	1.40	210.19	4.01	265.87	10.51	2.62	317.50	1937.18

## Data Availability

The original contributions presented in this study are included in the article. Further inquiries can be directed to the corresponding author.
